# MT2 receptor mediates melatonin-induced thermogenic program in human myoblasts: insights for circadian syndrome and diabesity treatment

**DOI:** 10.3389/fphar.2025.1633326

**Published:** 2025-07-08

**Authors:** Diego Salagre, Juan Sanjuán‐Hidalgo, Ehab Kotb Elmahallawy, Pedro P. Medina, Ahmad Agil

**Affiliations:** ^1^ Department of Pharmacology, BioHealth Institute Granada (IBs Granada), Neuroscience Institute (CIBM), School of Medicine, University of Granada, Granada, Spain; ^2^ Department of Biochemistry and Molecular Biology I, BioHealth Institute Granada (IBs Granada), GENYO, Centre for Genomics and Oncological Research: Pfizer/University of Granada/Andalusian Regional Government, Faculty of Sciences, University of Granada, Granada, Spain; ^3^ Department of Animal Health, School of Veterinary Medicine, Animal Health and Zoonosis Research Group (GISAZ), University of Córdoba, Córdoba, Spain; ^4^ Department of Zoonoses, Faculty of Veterinary Medicine, Sohag University, Sohag, Egypt

**Keywords:** human primary myoblast, melatonin, MT2, skeletal muscle, non-shivering thermogenesis, diabesity, Circadian syndrome

## Abstract

**Background:**

Melatonin is crucial for regulating circadian rhythms. Previous studies have demonstrated its ability to improve metabolic disorders, including obesity and associated diabetes (diabesity), in addition to its antioxidant, anti-inflammatory and anti-apoptotic properties. Recently, melatonin was shown to reduce obesity by increasing skeletal muscle (SKM) energy expenditure through non-shivering thermogenesis (NST). Small interfering RNAs (siRNAs) are powerful tools for inhibiting gene expression, enabling the analysis of gene functions and roles in molecular pathway activation. This study aimed to identify the receptor mediating melatonin’s pharmacological actions in SKM NST.

**Methods:**

Bioinformatics and protein-protein interaction (PPI) analyses were conducted. To examine the role of the melatonin receptor 2 (MT2) encoded by *MTNR1B*, we cultured human primary myoblasts and then silenced *MTNR1B* using siRNA transfection for 72 h, followed by 1 mM melatonin treatment for 24 h. Gene and protein expression were analyzed using semi-quantitative reverse transcriptase PCR and Western blotting respectively.

**Results:**

PPI analysis revealed *MTNR1B*’s strong association with diabetes, obesity, cancer, and circadian rhythm disorders, collectively known as circadian syndrome, and *MTNR1B*’s close interaction with thermogenic genes (*UCP1*, *PPARG*, and *PPARGC1A*). Silencing *MTNR1B* reduced the gene expression and inhibited the melatonin-induced upregulation of MT2 and NST-related proteins. Melatonin increased SERCA1/2, SLN, and Ca^2+^-dependent thermogenic pathway activation; however, these effects were abolished following *MTNR1B* knockdown.

**Conclusion:**

Our findings confirm that MT2 plays a key role in melatonin-driven SERCA-SLN uncoupling and the activation of the thermogenic program in SKM *via* the CaMKII/AMPK/PGC1α pathway upregulation. This study provides new insights into the molecular mechanisms underlying melatonin’s effects on thermogenesis and suggests potential melatonin-based therapeutic strategies against diabesity.

## Graphical Abstract

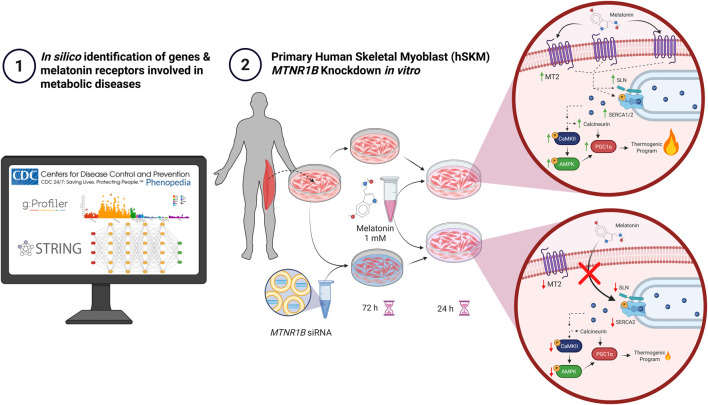

## 1 Introduction

Melatonin is recognized as a hormone primarily produced at night by the pineal gland (Navarro-Alarcón et al., 2014; Luo et al., 2024). Its primary function is to regulate circadian rhythms, essential for maintaining the body’s internal balance (Challet and Pévet, 2024). Beyond its well-established effects on the sleep-wake cycle, recent research highlights the broader pharmacological actions of melatonin, in particular its antioxidant, anti-inflammatory, anti-apoptotic, and energy balance regulation effects (Cipolla-Neto et al., 2014; Promsan and Lungkaphin, 2020). In particular, melatonin has demonstrated a significant thermogenic effect, which may help mitigate obesity, insulin resistance and hyperglycemia (Salagre et al., 2024b). This emerging evidence suggests that melatonin may play a key role in regulating body weight and metabolic homeostasis, making it a promising candidate for the treatment of metabolic disorders, including diabesity.

Despite advancements in healthcare and medicine, the increase in life expectancy over recent decades has not been accompanied by an improvement in the prevalence of obesity and metabolic diseases, the global prevalence of obesity and metabolic diseases continues to increase (Jura and Kozak, 2016; Zhang et al., 2023). Recent data indicates that one in eight people worldwide is obese, and approximately 43% of adults over the age of 18 are overweight (World Health Organization, 2022). This growing trend is concerning, as obesity is closely linked to an elevated higher risk of developing conditions such as type 2 diabetes and metabolic syndrome (World Health Organization, 2022). Metabolic syndrome is characterized by a set of disorders, such as central obesity, insulin resistance, hyperglycemia, hypertension, and dyslipidemia. In addition, metabolic syndrome together with main comorbidities including sleep disturbances, depression, steatohepatitis and cognitive dysfunction, was recently proposed to be called as “Circadian syndrome,” that has risen sharply in recent decades becoming a significant health threat in modern society (Wilkin and Voss, 2004; Saklayen, 2018; Zimmet et al., 2019). Addressing these interconnected conditions requires novel therapeutic approaches, and melatonin has garnered attention for its potential to mitigate these metabolic disturbances (Agil et al., 2011; Gao et al., 2024).

Preclinical studies, particularly in Zucker Diabetic Fatty (ZDF) rats, a widely used model for obesity and its associated type 2 diabetes, support the therapeutic potential of melatonin (Shiota and Printz, 2012; Capcarova and Kalafova, 2019). Acute melatonin administration in young male ZDF rats has shown that melatonin can reduce obesity and improve metabolic function (Agil et al., 2011; 2012; Navarro-Alarcón et al., 2014). These effects are partly attributed to the activation of brown adipose tissue and the “browning” of subcutaneous fat, which enhances the expression of the thermogenic protein uncoupling protein 1 (UCP1) and the regulator of thermogenesis protein peroxisome proliferator-activated receptor gamma (PPARγ) coactivator 1α (PGC-1α) (Jiménez-Aranda et al., 2013; Fernández Vázquez et al., 2018; Agil et al., 2021; Aouichat et al., 2022; Salagre et al., 2022). Recently, chronic administration of melatonin to adult male and female ZDF rats was found to enhance an important mechanism of thermogenesis in the skeletal muscle (SKM) *via* the uncoupling of the sarco-endoplasmic reticulum Ca^2+^-ATPase (SERCA) activity through sarcolipin (SLN) upregulation, mediated by Ca^2+^/calmodulin-dependent protein kinase II (CaMKII), AMP-activated protein kinase (AMPK), and PGC1α signaling. This process also increases mitochondrial biogenesis and thermogenic capacity, contributing to a metabolic improvement (Salagre et al., 2024b). Furthermore, chronic melatonin administration promotes a change in skeletal muscle fiber type from a glycolytic (fast twitch) to an oxidative (slow twitch) phenotype in the vastus lateralis (VL) muscle of obese diabetic rats (Salagre et al., 2025). This muscle fiber transition is linked to improved mitochondrial dynamics and autophagy (Salagre et al., 2023), further supporting the potential of melatonin as a therapeutic agent for obesity and related metabolic complications, including type 2 diabetes and diabesity (Salagre et al., 2024b).

To fully understand how melatonin exerts these effects, it is essential to identify the specific receptor that triggers the signaling cascade responsible for these responses (Nikolaev et al., 2021). Melatonin exerts its effects through several receptors and binding sites, including nuclear orphan nuclear receptor α (ROR-α), intracellular proteins such as calmodulin and quinone reductase 2, and plasma membrane melatonin receptors 1 (MT1) and 2 (MT2) (Slominski et al., 2012; Waly and Hallworth, 2015; Emet et al., 2016). Encoded by the *MTNR1A* and *MTNR1B* genes respectively, MT1 and MT2 belong to the G-protein-coupled receptor family and play a key role in the physiological and metabolic effects of melatonin (Slominski et al., 2012; Xu et al., 2020). Activation of these receptors triggers multiple intracellular signaling pathways, regulating thermogenesis, energy homeostasis and metabolic inflammation (Hong and Kim, 2024). The widespread distribution of these receptors across different tissues explains melatonin’s diverse effects. While MT1 and MT2 are expressed to a large extent in brain regions responsible for circadian control, such as the suprachiasmatic nucleus (Doghramji, 2007; Waly and Hallworth, 2015; Liu et al., 2016), they are also present in peripheral tissues. In adipose tissue, these receptors regulate brown adipose tissue activation and browning of white fat (Owino et al., 2016; Xu et al., 2020). In SKM, MT1 and MT2 modulate energy metabolism and mitochondrial biogenesis (Tan et al., 2016; Owino et al., 2019). Identifying the specific receptor responsible for melatonin’s thermogenic effects is essential for developing targeted therapies to combat metabolic disorders, including diabesity.

Clinical trials further support melatonin’s therapeutic potential in obesity and metabolic syndrome (Delpino and Figueiredo, 2021). Daily melatonin administration (5 mg for 2 months) improved dyslipidemia and blood pressure (Koziróg et al., 2011). Another trial in patients revealed that melatonin reduced oxidative stress (Morvaridzadeh et al., 2020), which is strongly associated with insulin resistance and abdominal fat accumulation that are key contributors to obesity and metabolic dysfunction (Azzeh et al., 2024). A recent systematic review and meta-analysis of 23 studies found that melatonin supplementation led to significant weight loss in 11 studies, with better outcomes observed at higher doses (8 mg/day) and longer treatment durations (48 weeks) (Delpino and Figueiredo, 2021). Despite these promising results, melatonin’s clinical efficacy remains variable, potentially due to genetic polymorphisms in *MTNR1B* and *MTNR1A* (Nikolaev et al., 2021; Li et al., 2023). These genetic differences may influence therapeutic responses to melatonin and other drugs used to treat type 2 diabetes, such as repaglinide (Wang et al., 2019). Further research involving diverse populations is necessary to fully elucidate these genetic influences and optimize melatonin-based therapies.

Thus, in the present work, we investigated whether *MTNR1B* mediates melatonin’s thermogenic effects in cultured human primary myoblasts *via* MT2 receptor activation. Our research aims to provide new insights into melatonin’s role as a thermogenic agent and its therapeutic potential for treating diabesity.

## 2 Materials and methods

### 2.1 Bioinformatics analysis

A PPI analysis was conducted, combined with a functional enrichment analysis. Initially, HuGE Navigator database (https://phgkb.cdc.gov/PHGKB/hNHome.action) was consulted, and through it, access to Phenopedia (https://phgkb.cdc.gov/PHGKB/startPagePhenoPedia.action) was obtained, a valuable resource for exploring phenotype-gene relationships. In Phenopedia, the following keywords were used: “obesity,” “diabetes,” “type 2 diabetes,” and “sleep disorders.” Duplicated genes were removed and a list of genes associated with different phenotypes was obtained.

The resulting list of genes was then subjected to functional enrichment analysis using g:Profiler (https://biit.cs.ut.ee/gprofiler/gost). G:Profiler is a widely used tool set for interpreting genes, protein, or genomic variant lists in terms of biological categories, including metabolic pathways and relevant cellular processes (Raudvere et al., 2019; Kolberg et al., 2023).

For data visualization, STRING (http://string-db.org/) was used, a tool for constructing protein-protein interaction networks. In the generated PPI network, each colored node represents a gene, while the edges between represent interactions between the corresponding proteins (Cheng et al., 2017; Reimand et al., 2019). The PPI network was constructed using a minimum interaction confidence score of 0.4. The resulting PPI network was imported into Cytoscape (version 3.10.3), an open-source software platform for visualizing and analyzing complex networks, such as PPIs (Majeed and Mukhtar, 2023; Zhang et al., 2025). Cytoscape enabled the evaluation of three topological parameters of interest: Degree, Betweenness, and Closeness.

### 2.2 Cell culture

Primary Human Skeletal Muscle Cells (hSKM) were purchased from ATCC (cat# PCS-950-010, American Tissue Culture Collection, Manassas, Virginia).

hSKM cells were seeded in flask at equal densities (5 × 10^3^ cells per cm^2^) in T-75 culture flasks with 10 mL of complete culture medium (CM) consisting of complete Dulbecco’s Modified Eagle’s Medium (DMEM, Gibco, Life Technologies, Spain) supplemented with L-glutamine (2 mM), 10% Fetal Bovine Serum (FBS, Gibco, Life Technologies, Spain) and 2% penicillin/streptomycin (P/S, Sigma, Spain) and cultured in an incubator at 37°C with a humidified atmosphere containing 5% CO_2_. The cell culture medium was replaced twice a week. Freshly isolated cells were grown in monolayer culture up to passage 4–5 at a seeding density of 5 × 10^3^ cells per cm^2^ at each passage.

Cells were divided into three experimental groups: untransfected cells (control group, C), cells transfected with a non-specific Scrambled siRNA (negative control group, siRNA C^−^), and cells transfected with siRNA targeting the *MTNR1B* gene (experimental group, *MTNR1B* siRNA).

### 2.3 *MTNR1B* knockdown

Once cells achieved 80% confluency, 1 × 10^6^ cells per well were plated in a 6-well plate. SiRNA specific for the human *MTNR1B* gene (ON-TARGETplus Human *MTNR1B* siRNA (SmartPool 5 nmol), Horizon, United Kingdom) and a non-targeting scrambled siRNA sequence (MISSION^®^ siRNA Universal Negative Control, Sigma-Aldrich, Spain) were purchased, with the following sequences provided (Table 1).

**TABLE 1:** List of siRNA sequences.

Name	Sequence
siRNA J-005670-05 MTNR1B	GCU​ACU​UAC​UGG​CUU​AUU​U
siRNA J-005670-06 MTNR1B	GUA​CGA​CCC​ACG​CAU​CUA​U
siRNA J-005670-07 MTNR1B	GGU​AAU​UUG​UUC​UUG​GUG​A
siRNA J-005670-08 MTNR1B	GAG​AAC​GGC​UCC​UUC​GCC​A
Scramble-Silencer-S	UAA​CGA​CGC​GAC​GAC​GUA​A
Scramble-Silencer-AS	UUA​CGU​CGU​CGC​GUC​GUU​A

The Scrambled siRNA mix (Silencer-S and -AS) and *MTNR1B* siRNA mix (J-005670-05, -06, -07, and -08) were prepared as a 10 µM stock solution and subsequently diluted in Opti-MEM reduced-serum medium (cat#31985062, Thermo Fisher Scientific, Spain) to the following working concentrations: 5 nM, 10 nM, 25 nM, 40 nM, 80 nM, 100 nM, and 120 nM following manufacturer’s instructions. These concentrations were selected to evaluate the knockdown efficiency and determine the optimal dosage. No cell death was observed after knockdown treatment. Lipofectamine RNAiMAX (cat#13778075, Thermo Fisher Scientific, Spain) was employed as the transfection reagent to facilitate transfection process and also diluted in Opti-MEM following manufacturer’s protocol. Lipofectamine RNAiMAX and siRNA mix were combined 1:1, incubated for 20 min at RT, and then added to cells maintained in DMEM supplemented only with 10% FBS as the transfection medium.

The knockdown was allowed to proceed for 72 h, ensuring adequate gene silencing. After this period, the transfection medium was carefully removed and replaced with CM to support subsequent experimental assays.

### 2.4 Melatonin treatment

Following 72 h post-knockdown, the *in vitro* treatment with melatonin is initiated. Melatonin was added to the cells at a concentration of 1 mM based on results of previous dose-response studies showing that in acute melatonin *in vitro* treatments, high doses are needed to reach significant effects in C2C12 myoblast (Kim et al., 2012; Chen et al., 2019). Once added, the melatonin treatment was maintained in the cells for 24 h.

### 2.5 Total RNA extraction and complementary DNA (cDNA) synthesis

To extract total RNA from hSKM cells, the RNeasy Mini Kit (cat#74104 and cat#74106, QIAGEN, Germany) was used according to the manufacturer’s instructions. Once isolated, RNA was quantified by spectrophotometric absorption at 230, 260, and 280 nm using a Nanodrop One/One (cat#ND-ONE-W, Thermofisher Scientific, Spain).

Subsequently, complementary DNA (cDNA) synthesis was conducted using 1.0 μg of RNA from each sample with the M-MLV Reverse Transcriptase Kit (ref#P0073), which includes M-MLV Reverse Transcriptase (200 U/μL) and Reaction Buffer (10x). The reaction also included RNase inhibitor (RiboLock RNase Inhibitor, Ref#EO0381, Thermofisher Scientific, Spain), Oligo d(T)16 (50 μM, ref#N8080128, Thermofisher Scientific, Spain), dNTP Set (100 mM, ref#R0181, Thermofisher Scientific, Spain), and nuclease-free water (Ref#P119 E, Promega Biotech Ibérica, S.L., Spain). The reverse transcription process was performed in a final reaction volume of 20 μL.

### 2.6 Gene expression analysis by reverse transcriptase semi-quantitative polymerase chain reaction (semi-quantitative RT-PCR)

For semi-quantitative RT-PCR, DreamTaq Polymerase Master Mix (cat#K1082, Thermofisher Scientific, Spain) was used following the manufacturer’s instructions. Primers used for amplifying the gene of interest (*MTNR1B*) were designed using the Primer-Blast platform from the National Center for Biotechnology Information (NCBI) and are listed below in Table 2.

**TABLE 2:** List of primers pair used in semi-quantitative RT-PCR.

Gene	Forward sequence (5′ → 3′)	Reverse sequence (5′ → 3′)
*B2M*	TGC​TGT​CTC​CAT​GTT​TGA​TGT​ATC​T	TCT​CTG​CTC​CCC​ACC​TCT​AAG​T
*MTNR1B*	GCT​GCC​CAA​CTT​CTT​TGT​GG	GAC​ACG​ACA​GCG​ATA​GGG​AG

Amplification was performed using a GeneAmp PCR System 2700 thermocycler (Applied Biosystems, Spain). The housekeeping gene B2M was used as an internal control. To confirm the results and validate cDNA quantification, standard curves were performed by amplifying first-strand cDNA for 25 to 40 cycles.

To further ensure RT-PCR quality, amplified products were separated on a 1.5% agarose gel containing SYBR Green I 10000 X (cat#S7563, Thermofisher Scientific, Spain), Orange DNA Loading Dye (cat#R0631, Thermofisher Scientific, Spain) as loading buffer, and TrackIt Ultra Low Range DNA Ladder (cat#10488023, Thermofisher Scientific, Spain) as DNA ladder. Densitometry analysis of bands was used to measure the gene expression as described in previous studies (Izzo et al., 2010; Wang, 2021).

### 2.7 Total protein extraction and protein expression analysis by Western Blot

Proteins were extracted from hSKM cells using the RIPA lysis buffer (50 mM Tris-HCl, 150 mM NaCl, 2 mM ethylenediaminetetraacetic acid (EDTA), and 0.1% sodium dodecyl sulfate (SDS). To improve the protein extraction process, 1% Triton X-100, 1% protease inhibitor cocktail, and 1% phosphatase inhibitor cocktail were added to the lysis buffer. Homogenization was performed using an ultrasonic homogenizer for two cycles of 10 s each. The homogenates obtained were subjected to centrifugation at a speed of 15,000 g for 15 min at 4°C. The supernatant was transferred to a new tube. Protein concentration was determined using the Bradford method, using bovine serum albumin (BSA) as a standard. A temperature of 4°C was maintained throughout the extraction process.

For the analysis and quantification of the extracted proteins, 30–50 µg of protein were separated by SDS-polyacrylamide gel electrophoresis (SDS-PAGE). After electrophoresis, the gels were transferred onto a nitrocellulose membrane (Bio-Rad Trans-Blot SD, Bio-Rad Laboratories, CA, United States). Following the transfer, the membranes were washed once with Phosphate Buffer Saline (PBS, 137 mM NaCl, 2.7 mM KCl, 10 mM Na_2_HPO_4_, and 1.8 mM KH_2_PO_4_, pH 7.4) (PBS) supplemented with 0.1% Tween-20 (PBS-T) for 10 min. The membranes were then blocked for 1 h at room temperature using a blocking solution (PBS-T supplemented with 5% BSA). After blocking, the membranes underwent a 15-min wash followed by three 10-min washes with PBS-T and were incubated overnight at 4°C with the primary antibody. The primary antibody was generated in goat against MT2 (cat#sc-13177, Santa Cruz Biotechnology, United States) and PGC1α (cat#SAB2500781, Sigma-Aldrich, Spain); in mice against Calcineurin (cat#H00005530-M03, Abnova, United States), SERCA2 (cat#S1439, Sigma-Aldrich, Spain), CaMKII (cat#SC-13141, Santa Cruz Biotechnology, United States), and P-CaMKII (cat#SC-32289, Santa Cruz Biotechnology, United States); and in rabbit against SLN (cat#MBS713457, MyBiosource, United States), SERCA1 (cat#SAB5701310, Sigma-Aldrich, Spain), AMPK (cat#SAB4502329, Sigma-Aldrich, Spain), and P-AMPK (cat#SAB4503754, Sigma-Aldrich, Spain); all diluted 1:1,000 in PBS-T with 10% blocking solution. After overnight incubation, the membranes were washed again for 15 min, followed by three 10-min washes with PBS-T to remove unbound primary antibodies. Anti-α-tubulin (cat#SC-5286; Santa Cruz Biotechnology, United States) and anti-GAPDH antibody (cat#SC-365062; Santa Cruz Biotechnology, United States) generated in mice was used as an internal loading control. The membranes were subsequently incubated at room temperature for 2.5 h with the respective anti-mouse (cat#MBS674947, MyBiosource, United States), anti-rabbit (cat#A16035, Thermofisher Scientific, Spain), and anti-goat (cat#A5420, Sigma-Aldrich, Spain) horseradish peroxidase (HRP)-conjugated secondary antibodies at a dilution of 1:2,000 in PBS-T with 10% blocking buffer. Following this incubation, the membranes were washed again for 15 min, followed by three 10-min washes with PBS-T to remove unbound secondary antibodies. Immunoreactive proteins were detected using the Pierce™ ECL Western Blotting Substrate kit (cat#32106, Thermofisher Scientific, Spain) according to the manufacturer’s instructions. Signal intensity was captured using the Image Station 4000MM Pro Molecular Imaging system (Kodak, United States) and quantitatively analyzed with ImageJ software version 1.33 (National Institutes of Health, Bethesda, MD, United States). The results were normalized to α-tubulin or GAPDH levels.

### 2.8 Statistical analysis

All experiments were repeated at least three times. Data are expressed as means ± standard deviation (SD). Means were compared between groups using one-way analysis of variance (ANOVA), adjusted by *post hoc* Tukey’s test. SPSS, version 22, for Windows (SPSS, Michigan, IL, United States) was used for data analyses. *P*-Value < 0.05 was considered statistically significant, and the levels of significance were labeled on the figures as follows: * *P* < 0.05 and ** *P* < 0.01, melatonin treated vs. non-treated groups; # *P* < 0.05 and ## *P* < 0.01, siRNA *MTNR1B* vs. Scrambled siRNA negative control (siRNA C^−^) transfected cells.

## 3 Results

### 3.1 *MTNR1B* is strongly associated with diabesity and circadian syndrome among melatonin receptors family

Through HuGE Navigator and Phenopedia, 4,573 genes associated with the desired phenotypes (obesity, diabetes, type 2 diabetes, intrinsic sleep disorders, sleep disorders, and circadian rhythm) were identified (Table 3). After removing duplicated genes, a total of 3,182 distinct genes associated with the mentioned phenotypes were obtained and considered for further analysis. The complete list of genes of interest analyzed can be found in Supplementary Table S1.

**TABLE 3:** Phenotypes and number of associated genes.

Phenotype	Genes
obesity	2211
diabetes	1,663
type 2 diabetes	140
sleep disorders, intrinsic	474
sleep disorders	72
sleep disorders, circadian rhythm	13

**FIGURE 1:**
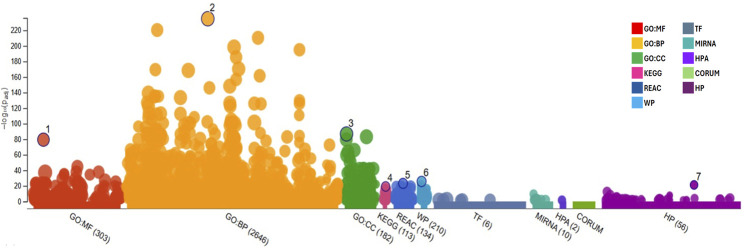
Functional enrichment graph obtained after results analysis from g:Profiler with the genes retrieved from Phenopedia for the desired phenotypes, representing all terms and their adjusted *P*-value [−log_10_(P_adj_)]. GO:MF, Molecular Function; GO:BP, Biological Process; GO:CC, Cellular Component; KEGG, Kyoto Encyclopedia of Genes and Genomes; REAC, Reactome Pathways; WP, WikiPathways; TF, Transcription Factors; MIRNA, MicroRNA; HPA, Human Protein Atlas; CORUM, Comprehensive Resource of Mammalian Protein Complexes; and HP, Human Phenotype.

This gene list was subjected to functional enrichment analysis using g:Profiler. As a result, it was observed that the set of introduced genes is associated with a wide variety of categories. Most of the introduced genes (2,646) are related to biological processes (GO: BP), followed by a smaller number of genes (303) linked to molecular activities (GO: MF), metabolic pathways related to diseases (210, WP), and cellular components (182; GO: CC). The results of the enrichment analysis can be found below in Figure 1, and the list with the most significant terms can be found in Table 4. Regarding the most important terms, the GO: BP terms such as “response to chemical,” “response to hormone,” and “response to stimulus” indicates that the introduced genes might be involved in cellular response to drugs and/or adaptation/defense mechanism to different external and internal stimulus like hormones and increased oxidative stress present in diabesity condition. Also, the term “temperature homeostasis” is found to be relevant in our gene list showing the close relationship between diabesity-associated genes and thermogenesis. The terms “lipid homeostasis” and “response to lipid” are important in diabesity, as remarked by its low *P*-adjusted value (P_adj_) after enrichment analysis of the studied genes. Moreover, the highlighted GO: CC terms “cell periphery,” “cell surface,” “plasma membrane” and “extracellular space” accompanied by the GO: MF terms “signaling receptor binding and activity” and “protein binding” suggest that these genes could play a key role in cellular communication and regulation, binding to different molecules in the plasma membrane region and placing greater importance on membrane receptors than nuclear or cytosolic ones in diabesity. Furthermore, REAC terms “signaling by GPCR” and “GPCR downstream signaling” suggest that the receptors located at the plasma membrane are coupled to G proteins regulation different cellular processes key for diabesity control. Membranal melatonin receptors MT1 and MT2 are two high-affinity G protein-coupled receptors which, together with the WP term “circadian rhythm genes” also highlighted for their importance after the functional enrichment analysis, showed the close relationship between melatonin, diabesity and circadian syndrome. In addition, the presence of terms related to metabolic pathways and metabolism regulation, such as “abnormality of metabolism/homeostasis“, “adipogenesis,” and “lipid and atherosclerosis,” implies that the analyzed genes may be involved in energy production and lipid storage. Finally, many terms related to diabesity complications such as meta-inflammation, cancer, and cardiovascular diseases were also found: “interleukin-4 and interleukin-13 signaling,” “cancer pathways,” “abnormal systemic blood pressure,” and “abnormal cardiovascular system physiology.”

**TABLE 4:** Functional enrichment analysis results for the selected gene list, highlighting the most significant terms with the highest *p*-values.

Source	Term name	*P*adj
GO:BP	Response to chemical	1.566 × 10^−259^
GO:BP	Response to stimulus	1.080 × 10^−241^
GO:BP	Cellular response to chemical stimulus	5.556 × 10^−214^
GO:BP	Cellular response to stimulus	3.234 × 10^−206^
GO:BP	Positive regulation of biological process	1.114 × 10^−176^
GO:BP	Response to endogenous stimulus	7.511 × 10^−162^
GO:BP	Response to hormone	7.277 × 10^−143^
GO:BP	Response to lipid	7.164 × 10^−116^
GO:BP	Cellular response to endogenous stimulus	2.245 × 10^−112^
GO:CC	Cell periphery	2.287 × 10^−99^
GO:CC	Extracellular space	4.575 × 10^−92^
GO:MF	Signaling receptor binding	1.066 × 10^−86^
GO:CC	Extracellular region	9.996 × 10^−84^
GO:CC	Plasma membrane	1.148 × 10^−78^
GO:MF	Protein binding	3.983 × 10^−70^
GO:CC	Cell surface	3.425 × 10^−65^
GO:BP	Temperature homeostasis	1.645 × 10^−58^
GO:MF	Signaling receptor activity	9.859 × 10^−49^
GO:BP	Lipid homeostasis	1.058 × 10^−38^
REAC	Signaling by GPCR	2.909 × 10^−33^
REAC	GPCR downstream signaling	1.113 × 10^−32^
KEGG	Neuroactive ligand-receptor interaction	1.393 × 10^−31^
KEGG	Lipid and atherosclerosis	4.147 × 10^−30^
WP	Adipogenesis	8.307 × 10^−30^
REAC	Interleukin-4 and Interleukin-13 signaling	2.335 × 10^−28^
WP	Cancer pathways	2.109 × 10^−25^
HP	Abnormality of metabolism/homeostasis	4.928 × 10^−25^
HP	Abnormal systemic blood pressure	4.125 × 10^−24^
HP	Abnormal cardiovascular system physiology	8.842 × 10^−22^
WP	Circadian rhythm genes	1.134 × 10^−20^
GO:CC	Transcription regulator complex	7.349 × 10^−20^
HP	Abnormal homeostasis	2.126 × 10^−18^
HP	Type II diabetes mellitus	6.331 × 10^−12^

P_adj_, Adjusted *P*-value for each category in the enrichment analysis; GO:MF, Molecular Function; GO:BP, Biological Process; GO:CC, Cellular Component; KEGG, Kyoto Encyclopedia of Genes and Genomes; REAC, Reactome Pathways; WP, WikiPathways; and HP, Human Phenotype.

**FIGURE 2:**
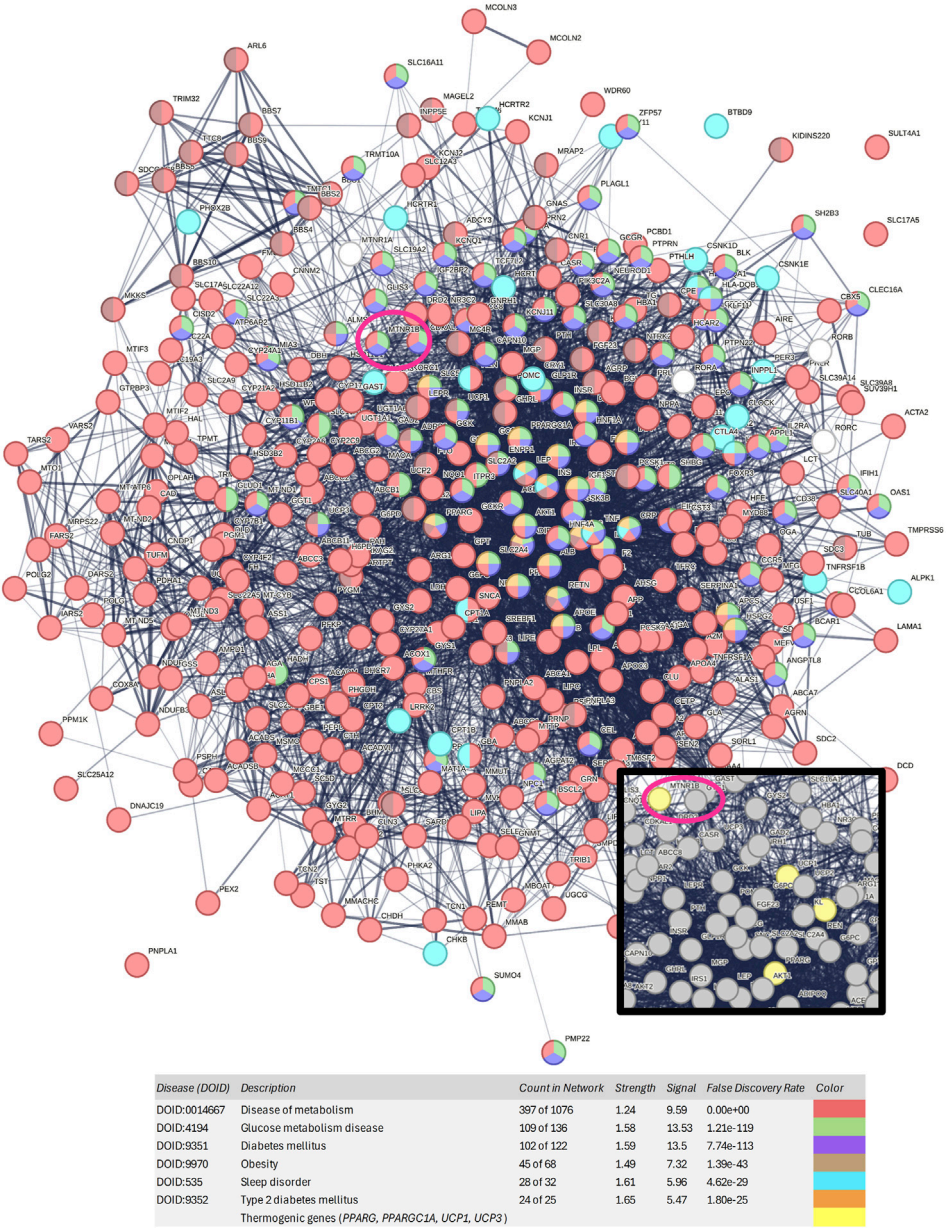
Protein-protein interaction network generated with the 397 genes associated with obesity, diabetes, type 2 diabetes, and sleep disorders. Nodes of red color represent diseases of metabolism, nodes of green color represent glucose metabolism diseases, nodes of purple color represent diabetes mellitus, nodes of brown color represent obesity, nodes of blue color represent sleep disorders, and nodes of orange color represent type 2 diabetes mellitus. *MTNR1B* is circled in dark pink. In the zoomed-in view, thermogenic genes can be observed in yellow color.

The gene list was inputted into the STRING platform to construct a protein-protein interaction (PPI) network. The network generated for the clusters “diabetes mellitus,” “disease of metabolism,” “glucose metabolism disease”, “obesity”, “sleep disorder”, and “type 2 diabetes mellitus” contained a total of 397 nodes and 5,315 edges. The average node degree was 26.8, meaning that, on average, each node is connected to more than 26 proteins within the network. In the visualization given in Figure 2, red nodes represent “disease of metabolism,” green nodes represent “glucose metabolism disease,” purple nodes represent “diabetes mellitus”, brown nodes represent “obesity”, blue nodes represent “sleep disorder”, and orange nodes represent “type 2 diabetes mellitus.”

Among the studied melatonin receptor genes, *MTNR1B MTNR1A*, and *RORA* showed strong association with both diabetes and obesity. Moreover, as shown in Figure 2 and Supplementary Table S2, *MTNR1B* is included in the purple, green, and red disease clusters, corresponding to the diabetes mellitus cluster, glucose metabolism disease cluster, and disease of metabolism cluster, respectively. However, *MTNR1A* and *RORA* are not included in the main disease cluster studied, showing that *MTNR1B*, among melatonin receptors, has a stronger association to metabolic diseases such as obesity and diabetes. Furthermore, *MTNR1B* was found to be located in close proximity to other thermogenic genes, such as *UCP1*, *PPARGC1A* and *PPARG*, suggesting its potential role in metabolic regulation and thermogenesis.

The PPI network was imported into Cytoscape. To identify critical targets within the network, three topological parameters were evaluated: Betweenness Centrality, Closeness Centrality, and Degree (Table 5).

**TABLE 5:** Main results of the topological parameters (degree, betweenness centrality, and closeness centrality) measured in genes of interest.

Gene	Degree	Betweenness centrality	Closeness centrality
*INS*	739	0.03394041935620997	0.5587939698492462
*AKT1*	731	0.026503660449040425	0.5583450492066679
*TNF*	719	0.02165030373341461	0.5562224889955982
*IL6*	707	0.018427701375878593	0.5518062723302898
*TP53*	659	0.03003541540673274	0.5498417721518987
*ALB*	633	0.02198878161278208	0.5449911782003529
*IL1B*	608	0.01076677883853121	0.5377176015473888
*STAT3*	535	0.009733774752207424	0.5301296720061022
*PPARG*	476	0.010678629406070313	0.5234419130107324
*LEP*	383	0.006610982139489241	0.5039883973894126
*APOE*	375	0.007926660804770879	0.5083196196745292
*IGF1*	371	0.0036288995813550163	0.5059144676979072
*GSK3B*	342	0.006168079486173016	0.5053626613343029
*SIRT1*	320	0.003795947165601392	0.5001799208348326
*PPARA*	306	0.004405973809048973	0.493432729854455
*CRP*	287	0.00214221025165658	0.48005525815921263
*PPARGC1A*	286	0.00463464469178503	0.4924712134632418
*ADIPOQ*	278	0.0029160842546203662	0.4881474978050922
*CAV1*	257	0.004227504372634952	0.4909058802754724
*GCG*	247	0.004304910630081138	0.4828065300451546
*IRS1*	243	0.0021617027100882353	0.4863540937718684
*HNF4A*	210	0.0027366688519582442	0.47898001378359756
*SLC2A4*	215	0.002646806536723463	0.47889750215331606
*UCP1*	125	5.19 × 10^−4^	0.44889391248183436
*UCP2*	120	5.69 × 10^−4^	0.4473049074818986
*RORC*	86	2.63 × 10^−4^	0.4139985107967238
*RORA*	74	3.56 × 10^−4^	0.4263803680981595
*MTNR1B*	68	7.25 × 10^−4^	0.416791604197901
*UCP3*	61	9.91 × 10^−5^	0.4079835632521279
*RORB*	37	1.35 × 10^−4^	0.3716577540106952
*MTNR1A*	26	8.35 × 10^−5^	0.39143094841930115

Degree indicates the number of direct connections a node has with other nodes in the network, representing its level of interaction. *PPARG*, *PPARGC1A*, and *PPARA* (peroxisome proliferator-activated receptor alpha) present high degrees, consolidating their relevance as pivotal points in the metabolic network (476, 286, and 306 respectively). Genes such as insulin (*INS*), albumin (*ALB*), interleukin 6 (*IL6*), apolipoprotein E (*APOE*), leptin (*LEP*), protein kinase B (*AKT1*), tumor necrosis factor (*TNF)*, signal transducer and activator of transcription 3 (*STAT3*), tumor protein 53 *(TP53)* and sirtuin 1 *(SIRT1)* have high degrees, indicating that they are highly involved in a well-connected network (739, 633, 707, 375, 383, 731, 719, 535, 659, and 320 respectively). The degree of *MTNR1B* is relatively low compared to other genes (68), but it indicates that it has some interactions with other nodes in the network. Although it is not as interconnected as the more central genes, its presence in the PPI network suggests that it may have an impact on specific metabolic processes, such as the circadian regulation of metabolism and its influence on insulin secretion rhythms and other metabolic processes. In comparison to *MTNR1B*, *MTNR1A* has a much lower Degree value (26), reinforcing that it is *MTNR1B*, not *MTNR1A*, the target gene. In contrast, *RORA* and *RORC* exhibit slightly higher values (74 and 86, respectively) than *MTNR1B*, suggesting a more generalized, possibly as intermediate in multiple pathways, rather than a specific function in a particular molecular pathway like *MTNR1B*.

Betweenness Centrality measures the ability of a node to function as a mediator in communication between other nodes in the network. Proteins with high values for this parameter are essential for signal integration and can control the flow of metabolic information between different nodes. The genes *PPARG*, *PPARGC1A*, and *PPARA* show high values for this parameter (0.011, 0.005, and 0.004 respectively), indicating that they are key intermediaries in the network interactions. Other genes with elevated values include *INS*, *ALB*, *APOE* and *LEP*, all of which are involved in the regulation of glucose metabolism, lipid signaling, and the control of energy balance (0.034, 0.022, 0.008, and 0.007 respectively). Elevated values are also observed for the genes *AKT1*, *TNF*, *IL6*, *TP53*, interleukin 1 beta (*IL1B*), *STAT3*, and *SIRT1*, which are primarily involved in the regulation of inflammation and the cellular response, potentially influencing energy metabolism (0.027, 0.022, 0.018, 0.030, 0.011, 0.009, and 0.004 respectively). The Betweenness Centrality value for *MTNR1B* is low (7.25 × 10^−4^), suggesting that although this gene may be relevant in some network interactions, it does not occupy a critical position in signal transmission compared to other genes. This does not mean it is unimportant, but rather that its influence may be mediated more indirectly. Compared to *MTNR1A* (8.35 × 10^−5^), the Betweenness Centrality value of *MTNR1B* is almost 10 times higher. Furthermore, *RORA* and *RORC* show lower values (3.56 × 10^−4^ and 2.63 × 10^−4^, respectively) than *MTNR1B*, reinforcing the idea that *MTNR1B* plays a more significant role in the interactions within the network, although in a less central manner.

Closeness Centrality reflects how close a node is to all others in the network, indicating its efficiency in transmitting information to other proteins. If the distance between two nodes is smaller than the distance between other nodes, then information will pass more quickly between these nodes. Therefore, these nodes may have an influential role in the network. *PPARG*, *PPARGC1A*, and *PPARA*, similar to the previous parameter, show high values, suggesting that these proteins are strategically positioned to influence the network centrally, consistent with their key roles in metabolic regulation and lipid metabolism (0.523, 0.492, and 0.493, respectively). *UCP1* and uncoupling protein 2 (*UCP2)* have intermediate values (5.19 × 10^−4^ and 5.69 × 10^−4^ respectively), which may reflect their involvement in specific processes within energy metabolism, such as thermogenesis. *INS* and *ALB* also have elevated values (0.559 and 0.545 respectively), further emphasizing their importance in regulating energy balance and glucose, and the same occurs with *AKT1*, *TNF*, *IL6*, *IL1B*, *STAT3*, *SIRT1*, and *TP53* highlighting their importance (0.558, 0.556, 0.552, 0.538, 0.530, 0.500, and 0.550 respectively). *MTNR1B* does not show high values for Closeness Centrality, indicating that it does not affect a large number of genes in the network (0.418). This also suggests that *MTNR1B* may have a more specialized and localized role in regulating specific processes. *MTNR1A* has an even lower value (0.391), indicating that its influence on the network is even more limited. *RORA* and *RORC* have values (0.426 and 0.414, respectively) similar to *MTNR1B*, which could indicate that all are equally involved in the network, potentially working together in related processes, although they have different roles.

### 3.2 Dose-effect curve

**FIGURE 3:**
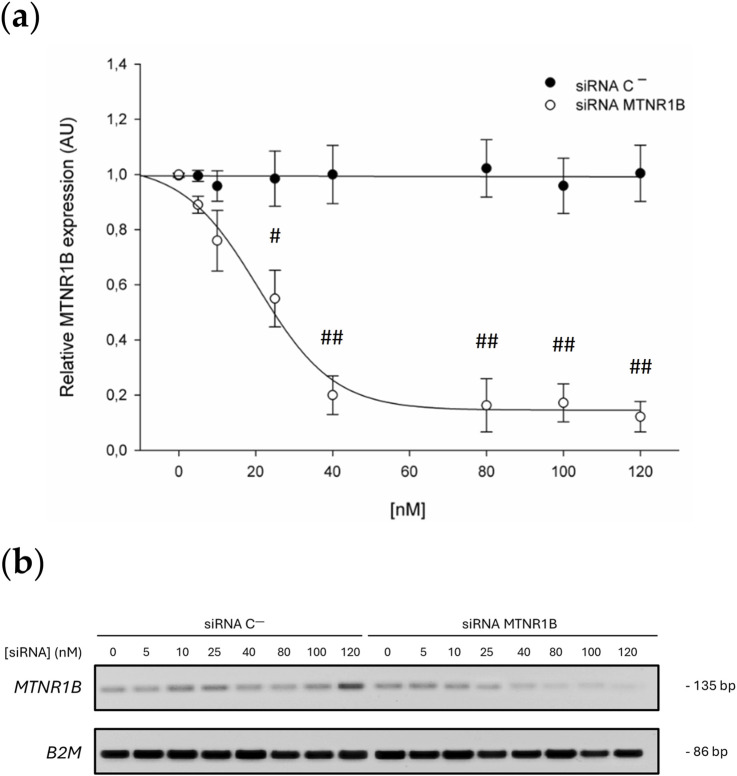
**(a)** Dose-effect curve. Black dots correspond to the *MTNR1B* expression in Scrambled siRNA negative control (siRNA C^−^)-transfected cells, and white dots correspond to the relative *MTNR1B* expression in siRNA *MTNR1B*-transfected cells. **(b)** Agarose gel electrophoresis of representative RT-PCR products. All values are obtained from a densitometry analysis of mRNA expression and expressed as mean ± SD of three independent experiments in triplicate (# *P* < 0.05 and ## *P* < 0.01, siRNA *MTNR1B* vs. Scrambled negative control).

A clear dose-dependent relationship was observed in the expression of the *MTNR1B* gene when the concentration of siRNA increased. Cells transfected with siRNA against *MTNR1B* significantly reduced the expression of MT2 starting from 25 nM siRNA concentration compared to Scrambled siRNA negative control (siRNA C^−^) transfected cells. 25 nM siRNA *MTNR1B*-transfected cells decreased the *MTNR1B* expression compared to cells transfected with siRNA C^−^ (siRNA C^−^ 25 nM, 0.985 ± 0.154 vs siRNA *MTNR1B* 25 nM, 0.579 ± 0.133, *P* < 0.05). Increased concentration of siRNA *MTNR1B* decreased the *MTNR1B* gene expression in transfected cells (40 nM, 0.203 ± 0.071; 80 nM, 0.163 ± 0.096; 100 nM, 0.172 ± 0.069; 120 nM, 0.122 ± 0.055) compared to siRNA C^−^transfected cells (40 nM, 1.015 ± 0.134; 80 nM, 1.038 ± 0.123; 100 nM, 0.989 ± 0.098; 120 nM, 1.002 ± 0.145; *P* < 0.01; Table 6; Figure 3a). With siRNA concentration greater than 120 nM, a plateau effect was reached, indicating that maximum possible efficacy in gene silencing was achieved (Figure 3a). The dose-effect curve analysis determined that the half-maximal inhibitory concentration (IC50) of the siRNA was 28.20 nM, while the 70% inhibitory concentration (IC70) was 39.83 nM. This confirmed that at a siRNA concentration of approximately 40 nM, 70% or more inhibition of mRNA expression was achieved, indicating that the silencing was effectively performed. The knockdown effects on *MTNR1B* expression can also be observed in the agarose gel electrophoresis of RT-PCR products shown in Figure 3b. From the obtained dose-effect values shown in Table 6, the dose-effect curve was fitted using the following sigmoidal equation, where the estimated parameter values were a = 0.8890, b = −9.8469, x_0_ = 20.6369, and y_0_ = 0.1455:

$$f\left(x\right)=y_{0}+\frac{a}{1+e^{‐\frac{x\;‐\;x_{0}}{b}}\;}$$

**TABLE 6:** *In vitro* dose-effect curve data. All values are expressed as mean ± SD of three independent experiments in triplicate. A one-way ANOVA followed by Tukey’s *post hoc* test was performed for statistical analysis (# *P* < 0.05 and ## *P* < 0.01, siRNA *MTNR1B* vs. Scrambled negative control).

[nM]	Relative MTNR1B expression (AU)	
	siRNA C^−^	siRNA MTNR1B
0	1.000 ± 0.007	1.000 ± 0.012
5	0.995 ± 0.056	0.891 ± 0.031
10	0.958 ± 0.100	0.757 ± 0.139
25	0.985 ± 0.154	0.579 ± 0.133 #
40	1.015 ± 0.134	0.203 ± 0.071 ##
80	1.038 ± 0.123	0.163 ± 0.096 ##
100	0.989 ± 0.098	0.172 ± 0.069 ##
120	1.002 ± 0.145	0.122 ± 0.055 ##
y_0_ = Max Effect	—	0.1455
IC_50_ (28.20 nM)	—	0.4273
IC_70_ (39.83 nM)	—	0.2964

### 3.3 Effect of melatonin on MT2 expression

**FIGURE 4:**
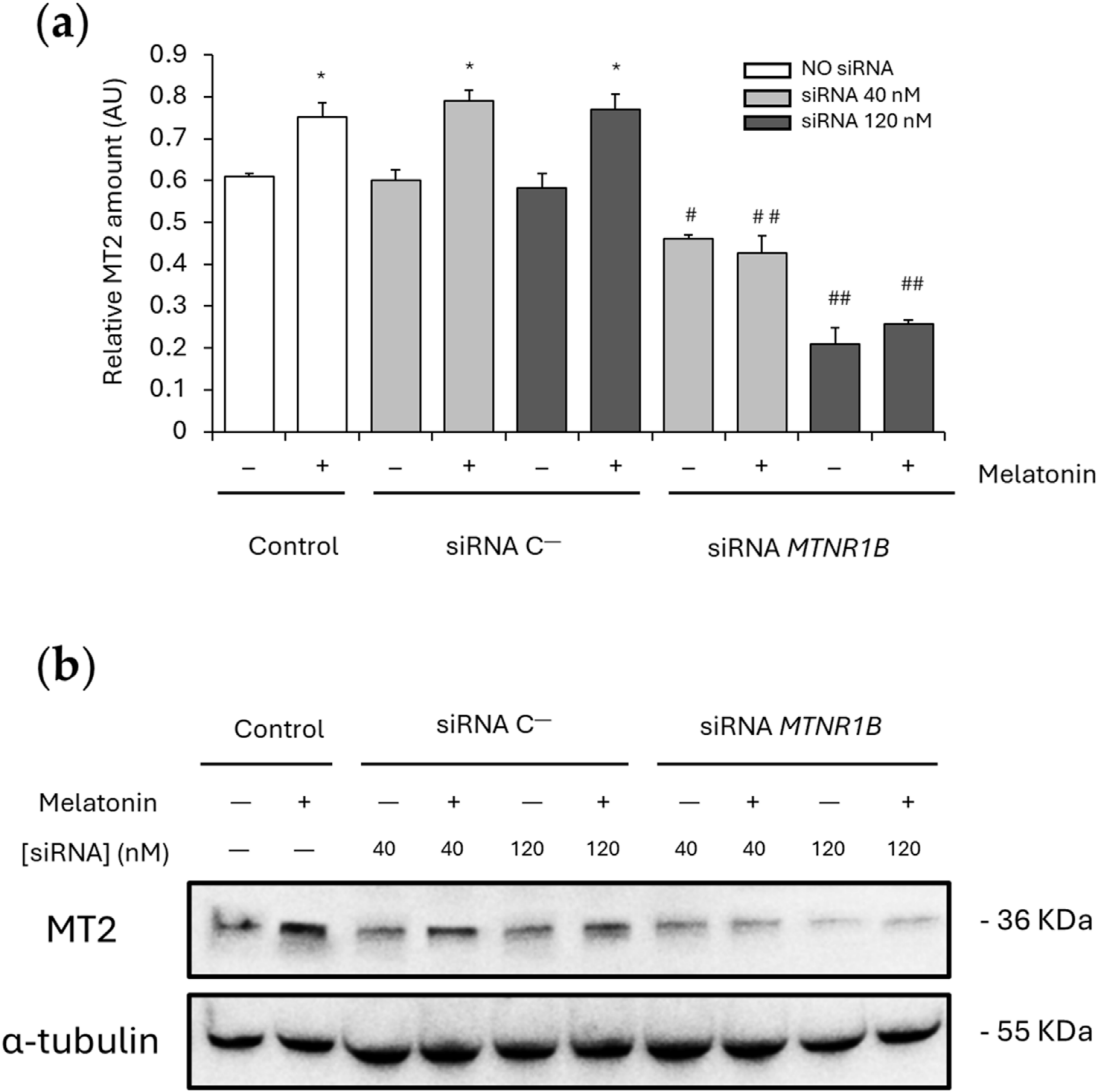
Effect of melatonin on MT2 expression. **(a)** Densitometry quantification of MT2 amount in unsilenced and *MTNR1B*-silenced human myoblast. **(b)** Representative blot of MT2 expression. α-tubulin was used as loading control. All values are expressed as mean ± SD of three independent experiments in triplicate. A one-way ANOVA followed by Tukey’s *post hoc* test was performed for statistical analysis (* *P* < 0.05, Melatonin treated vs. Non-treated; # *P* < 0.05 and ## *P* < 0.01, siRNA *MTNR1B* vs. Scrambled negative control, siRNA C^−^).

No significant differences were found between the MT2 expression values obtained for cells transfected with siRNA C^−^ (40 nM, 0.600 ± 0.025; 120 nM, 0.583 ± 0.033) and the control untransfected cells (0 nM, 0.610 ± 0.007). After melatonin treatment, a significant increase in MT2 expression was observed in untransfected and siRNA C^−^transfected cells (0 nM, 0.751 ± 0.035; 40 nM, 0.790 ± 0.025; 120 nM, 0.769 ± 0.037; *P* < 0.05), demonstrating that MT2 expression is upregulated by melatonin. Knockdown of *MTNR1B* mRNA yielded a significant decrease of MT2 protein expression compared to siRNA C^−^transfected cells (siRNA *MTNR1B* 40 nM, 0.460 ± 0.009, and siRNA *MTNR1B* 120 nM, 0.209 ± 0.038; *P* < 0.05 and *P* < 0.01, respectively; Figure 4a). While, at the mRNA level, a gene expression inhibition of 70% or more was achieved with a siRNA *MTNR1B* concentration of approximately 40 nM (Figure 3a), at the protein level, this was achieved at a concentration of 120 nM (Figure 4a). In siRNA *MTNR1B*-transfected cells, the addition of melatonin did not increase MT2 expression compared to melatonin untreated cells at both concentrations studied. Moreover, melatonin-treated and untreated siRNA *MTNR1B*-transfected cells presented lower MT2 protein levels than melatonin-treated untransfected and siRNA C^−^transfected cells (40 nM, 0.427 ± 0.042; 120 nM, 0.256 ± 0.010; *P* < 0.01; Figure 4a). These results show that *MTNR1B* knockdown effectively reduces MT2 melatonin receptor expression at the protein level as well and that melatonin treatment does not alter MT2 expression when the receptor is silenced. The effects of melatonin on MT2 expression are also shown in Figure 4b.

### 3.4 MT2 modulates melatonin’s effects on SERCA and SLN expression

**FIGURE 5:**
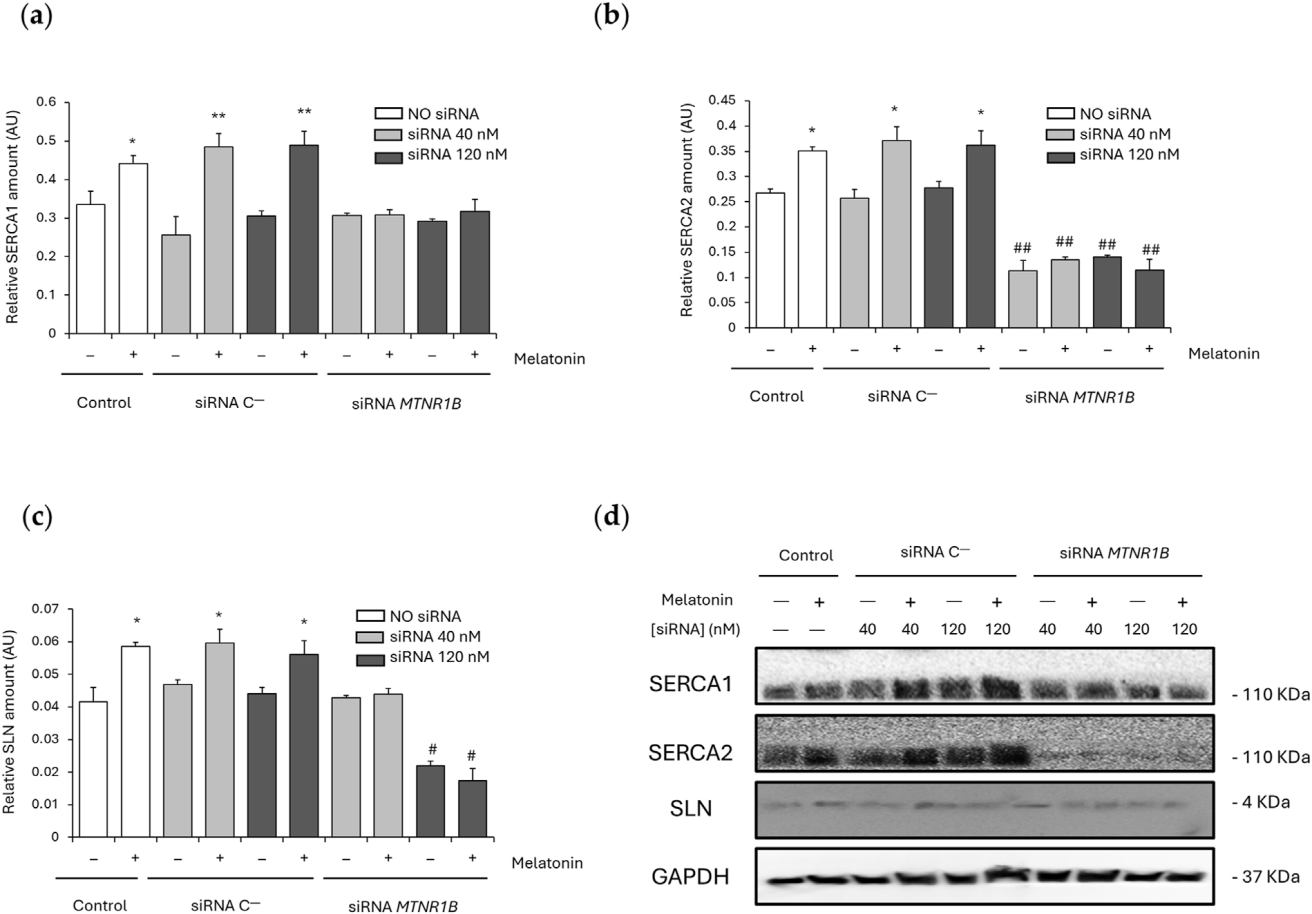
Effect of melatonin on SERCA/SLN uncoupling. **(a)** Densitometry quantification of SERCA1 amount in unsilenced and *MTNR1B*-silenced human myoblast. **(b)** Densitometry quantification of SERCA2 amount in unsilenced and *MTNR1B*-silenced human myoblast. **(c)** Densitometry quantification of SLN amount in unsilenced and *MTNR1B*-silenced human myoblast. **(d)** Representative blot of SERCA1, SERCA2, and SLN expression in unsilenced and *MTNR1B*-silenced human myoblast. GAPDH was used as loading control. All values are expressed as mean ± SD of three independent experiments in triplicate. A one-way ANOVA followed by Tukey’s *post hoc* test was performed for statistical analysis (* *P* < 0.05 and ** *P* < 0.01, Melatonin treated vs. Non-treated; # *P* < 0.05 and ## *P* < 0.01, siRNA *MTNR1B* vs. Scrambled negative control, siRNA C^−^).

SLN is responsible for Ca^2+^-dependent muscle thermogenesis through SERCA activity uncoupling. For this reason, SERCA1, SERCA2, and SLN expression were also analyzed. No significant differences were found between the SERCA1/2 and SLN expression values obtained for cells transfected with siRNA C^−^ (SERCA1 40 nM, 0.26 ± 0.048 and 120 nM, 0.31 ± 0.013; SERCA2 40 nM, 0.26 ± 0.018 and 120 nM, 0.28 ± 0.012; and SLN 40 nM, 0.047 ± 0.0014 and 120 nM, 0.044 ± 0.0019) and the control untransfected cells (SERCA1, 0.33 ± 0.035; SERCA2, 0.27 ± 0.008; and SLN, 0.042 ± 0.0044). After melatonin treatment, untransfected and siRNA C^−^transfected cells showed a significant increase of SERCA1 (untransfected, 0.44 ± 0.022; *P* < 0.05; 40 nM, 0.48 ± 0.035 and 120 nM, 0.49 ± 0.037; *P* < 0.01; Figure 5a), SERCA2 (untransfected, 0.35 ± 0.008; 40 nM, 0.37 ± 0.028; and 120 nM, 0.36 ± 0.029; *P* < 0.05; Figure 5b) and SLN expression (untransfected, 0.059 ± 0.0013; 40 nM, 0.060 ± 0.0043; and 120 nM, 0.056 ± 0.0042; *P* < 0.05; Figure 5c), suggesting that melatonin promotes SERCA/SLN uncoupling by increasing the expression of these proteins.

*MTNR1B* knockdown at 40 nM showed no differences in SERCA1 (0.31 ± 0.006) and SLN (0.043 ± 0.0008) expression compared to untransfected and siRNA C^−^transfected cells, and SERCA1 expression was also unchanged after *MTNR1B* silencing at 120 nM (0.29 ± 0.006). However, SERCA2 expression was observed to be lowered in siRNA *MTNR1B* transfected cells compared to untransfected and siRNA C^−^transfected cells at both concentrations (40 nM, 0.11 ± 0.021; and 120 nM, 0.14 ± 0.003; *P* < 0.01; Figure 5b) and SLN expression was also decreased in 120 nM siRNA *MTNR1B* transfected cells (0.022 ± 0.0015; *P* < 0.05; Figure 5c). This indicates that an inhibition of more than 50% of MT2 receptor expression is essential for a significant decrease in SLN levels. After melatonin treatment, siRNA *MTNR1B*-transfected cells at both concentrations showed no differences in SERCA1/2 and SLN expression, suggesting that MT2 functionality and expression are required for increased melatonin-mediated SERCA/SLN uncoupling. The effects of melatonin on SERCA1, SERCA2, and SLN expressions are shown in blots from Figure 5d.

### 3.5 MT2 modulates melatonin’s effects on Ca^2+^-dependent thermogenic pathway activation

SERCA/SLN uncoupling usually leads to increased cytosolic Ca^2+^ levels, which can activate Ca^2+^-dependent pathways *via* CaMKII and AMPK phosphorylation and/or calcineurin overexpression. This, in turn, promotes the upregulation of mitochondrial biogenesis and thermogenesis regulatory proteins such as PGC1α.

**FIGURE 6:**
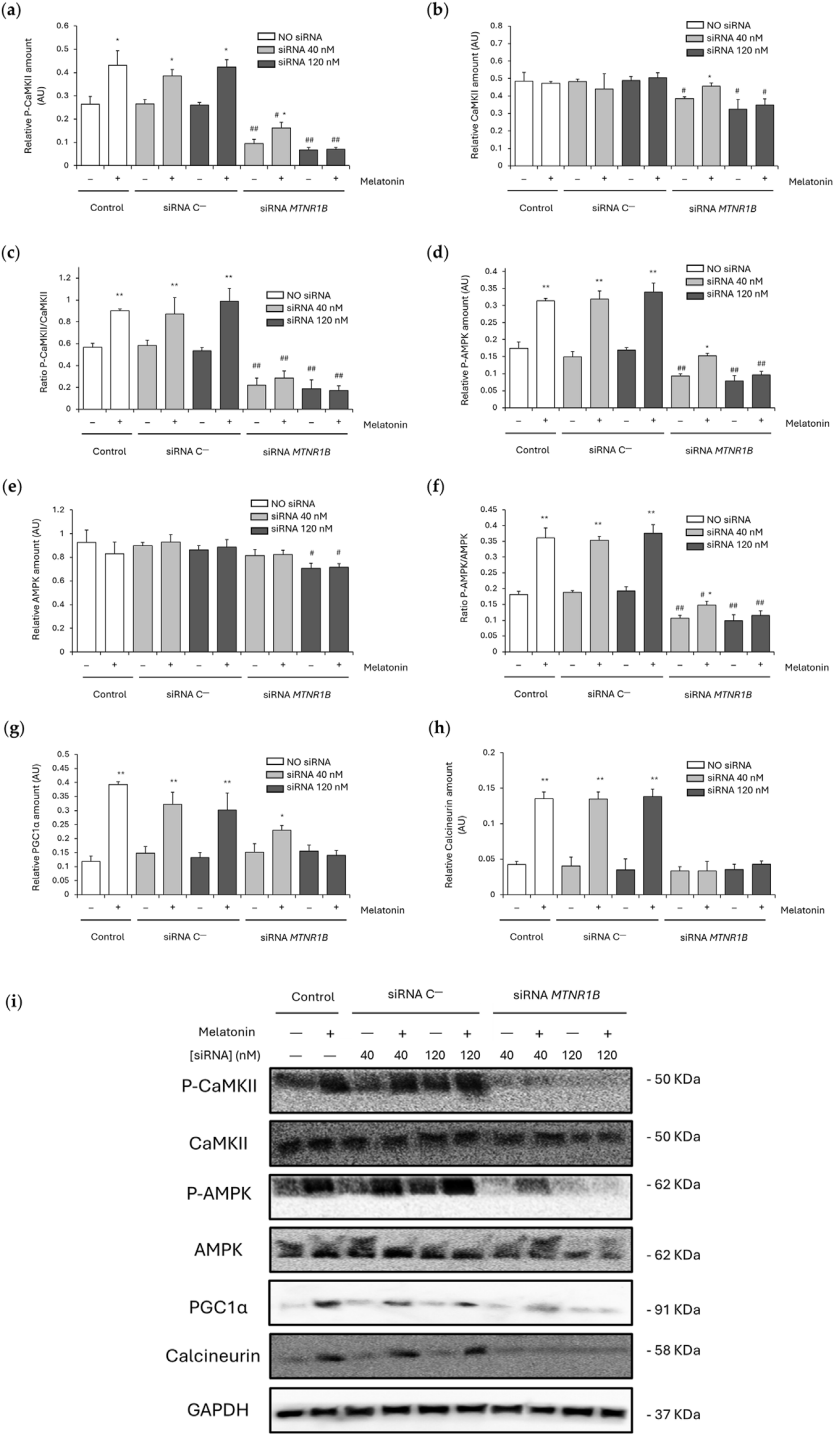
Effect of melatonin on the activation of Ca^2+^-dependent thermogenic pathway. **(a)** Densitometry quantification of P-CaMKII amount in unsilenced and *MTNR1B*-silenced human myoblast. **(b)** Densitometry quantification of CaMKII amount in unsilenced and *MTNR1B*-silenced human myoblast. **(c)** Ratio of P-CaMKII/CaMKII expression (activated (phosphorylated)/total) in unsilenced and *MTNR1B*-silenced human myoblast. **(d)** Densitometry quantification of P-AMPK amount in unsilenced and *MTNR1B*-silenced human myoblast. **(e)** Densitometry quantification of AMPK amount in unsilenced and *MTNR1B*-silenced human myoblast. **(f)** Ratio of P-AMPK/AMPK expression (activated (phosphorylated)/total) in unsilenced and *MTNR1B*-silenced human myoblast. **(g)** Densitometry quantification of PGC1α amount in unsilenced and *MTNR1B*-silenced human myoblast. **(h)** Densitometry quantification of Calcineurin amount in unsilenced and *MTNR1B*-silenced human myoblast. **(i)** Representative blot of P-CaMKII, CaMKII, P-AMPK, AMPK, PGC1α, and Calcineurin expression in unsilenced and *MTNR1B*-silenced human myoblast. GAPDH was used as loading control. All values are expressed as mean ± SD of three independent experiments in triplicate. A one-way ANOVA followed by Tukey’s *post hoc* test was performed for statistical analysis (* *P* < 0.05 and ** *P* < 0.01, Melatonin treated vs. Non-treated; # *P* < 0.05 and ## *P* < 0.01, siRNA *MTNR1B* vs. Scrambled negative control, siRNA C^−^).

No differences were observed between untransfected and siRNA C^−^-transfected cells in P-CaMKII (untransfected, 0.26 ± 0.033; 40 nM, 0.27 ± 0.018 and 120 nM, 0.26 ± 0.012), CaMKII (untransfected, 0.48 ± 0.051; 40 nM, 0.48 ± 0.013 and 120 nM, 0.49 ± 0.022), P-AMPK (untransfected, 0.17 ± 0.018; 40 nM, 0.15 ± 0.015 and 120 nM, 0.17 ± 0.007), AMPK (untransfected, 0.93 ± 0.11; 40 nM, 0.90 ± 0.03 and 120 nM, 0.86 ± 0.04), PGC1α (untransfected, 0.12 ± 0.019; 40 nM, 0.15 ± 0.024 and 120 nM, 0.13 ± 0.018) and calcineurin expression (untransfected, 0.043 ± 0.004; 40 nM, 0.040 ± 0.012 and 120 nM, 0.035 ± 0.015). The ratios P-CaMKII/CaMKII and P-AMPK/AMPK also remains unaltered in untransfected (P-CaMKII/CaMKII, 0.57 ± 0.03; and P-AMPK/AMPK, 0.18 ± 0.010) and siRNA C^−^-transfected cells at 40 nM (P-CaMKII/CaMKII, 0.58 ± 0.05; and P-AMPK/AMPK, 0.19 ± 0.006) and 120 nM (P-CaMKII/CaMKII, 0.54 ± 0.03; and P-AMPK/AMPK, 0.19 ± 0.014). Melatonin enhanced P-CaMKII and P-AMPK expression in untransfected (P-CaMKII, 0.43 ± 0.063; *P* < 0.05; and P-AMPK, 0.31 ± 0.007; *P* < 0.01) and siRNA C^−^transfected cells at 40 nM (P-CaMKII, 0.39 ± 0.028; *P* < 0.05; and P-AMPK, 0.32 ± 0.023; *P* < 0.01) and 120 nM (P-CaMKII, 0.42 ± 0.032; *P* < 0.05; and P-AMPK, 0.34 ± 0.025; *P* < 0.01) as shown in Figures 6a,d. However, CaMKII and AMPK levels were maintained unchanged after melatonin treatment (Figures 6b,e respectively). Therefore, melatonin increased in untransfected and siRNA C^−^-transfected cells the ratio P-CaMKII/CaMKII (untransfected, 0.90 ± 0.02; 40 nM, 0.87 ± 0.15 and 120 nM, 0.99 ± 0.12; *P* < 0.01; Figure 6c) and P-AMPK/AMPK (untransfected, 0.36 ± 0.032; 40 nM, 0.35 ± 0.012 and 120 nM, 0.38 ± 0.027; *P* < 0.01; Figure 6f). Furthermore, as shown in Figures 6g,h, melatonin promoted PGC1α and calcineurin expression in untransfected (PGC1α, 0.39 ± 0.010; and calcineurin, 0.135 ± 0.009; *P* < 0.01) and siRNA C^−^-transfected cells (40 nM: PGC1α, 0.32 ± 0.042; calcineurin, 0.135 ± 0.010; and 120 nM: PGC1α, 0.30 ± 0.061; calcineurin, 0.138 ± 0.010; *P* < 0.01).

Knockdown of *MTNR1B* at both concentrations lowered the levels of P-CaMKII (40 nM, 0.10 ± 0.018; and 120 nM, 0.07 ± 0.010; *P* < 0.01; Figure 6a), CaMKII (40 nM, 0.38 ± 0.010; and 120 nM, 0.33 ± 0.055; *P* < 0.05; Figure 6b) and P-AMPK (40 nM, 0.09 ± 0.007; and 120 nM, 0.08 ± 0.016; *P* < 0.01; Figure 6d), also lowering the ratios P-CaMKII/CaMKII (40 nM, 0.22 ± 0.06; and 120 nM, 0.19 ± 0.08; *P* < 0.01; Figure 6c) and P-AMPK/AMPK (40 nM, 0.11 ± 0.009; and 120 nM, 0.10 ± 0.020; *P* < 0.01; Figure 6f). No differences at 40 nM *MTNR1B* gene knockdown were observed in AMPK (0.81 ± 0.05), PGC1α (0.15 ± 0.031) and calcineurin expression (0.033 ± 0.006), nor expression differences were found in these last two proteins at 120 nM (PGC1α, 0.16 ± 0.022; and calcineurin, 0.036 ± 0.007; Figures 6g,h respectively) compared to untransfected and siRNA C^−^transfected cells. However, AMPK expression was observed to be lowered in 120 nM siRNA *MTNR1B* transfected cells (0.71 ± 0.04; *P* < 0.05), as shown in Figure 6e. After melatonin treatment, siRNA *MTNR1B*-transfected cells at 40 nM showed increased levels of P-CaMKII (0.16 ± 0.025; *P* < 0.05; Figure 6a), CaMKII (0.46 ± 0.019; *P* < 0.05; Figure 6b), P-AMPK (0.15 ± 0.007; *P* < 0.05; Figure 6d), Ratio P-AMPK/AMPK (0.15 ± 0.012; *P* < 0.05; Figure 6f), and PGC1α (0.23 ± 0.015; *P* < 0.05; Figure 6g). However, 120 nM siRNA *MTNR1B*-transfected cells presented no differences in protein expression and either in both ratio levels after melatonin treatment, suggesting that more than 50% of MT2 receptor expression is essential for an effective gene knockdown and that MT2 plays a key role in melatonin activation of the Ca^2+^-dependent thermogenic pathway through CaMKII/AMPK/PGC1α. The effects of melatonin on CaMKII, AMPK, PGC1α, and calcineurin expression are shown in blots from Figure 6i.

## 4 Discussion

Regarding the bioinformatic study, the functional analysis of genes associated with obesity, type 2 diabetes, and sleep disorders identified 397 key genes using advanced tools such as g: Profiler, STRING and Cytoscape. The construction of a protein-protein interaction (PPI) network revealed a complex interaction among these genes, encompassing 397 nodes and 5,315 edges, and an average node degree of 26.8. This robust and densely interconnected network underscores the existence of shared biological mechanisms underlying these phenotypes. A key finding was the strong association of the *MTNR1B* gene with metabolic phenotypes, including type 2 diabetes and obesity. Notable, *MTNR1B* gene showed a more prominent association with clusters related to metabolic diseases, including glucose metabolism, compared to related genes such as *MTNR1A* or *RORA* (Lyssenko et al., 2009). *RORA* and *RORC* exhibit higher Degree and Betweenness values than *MTNR1B* in the PPI network, suggesting a higher centrality of these genes in the studied pathway maybe acting as downstream signal transducer intermediary or second messenger, and in agreement with recent studies indicating that these genes act more as mediators of melatonin’s effects rather than as direct receptors. Furthermore, it has been discovered that they potentially function independently of melatonin-induced signaling (Ma et al., 2021). In contrast, *MTNR1B*, which encodes the MT2 receptor, is considered a key player in mediating melatonin’s direct impact on metabolic processes. This suggests a unique and direct role for *MTNR1B* in metabolic regulation. Moreover, melatonin could potentially bind to nuclear receptors that have yet to be identified (Panmanee et al., 2025). This further highlights the importance of *MTNR1B*, which is considered an active melatonin receptor, and its direct impact on metabolic processes. *MTNR1B* showed low topological values in the PPI network, suggesting that its role does not rely on a central position as an intermediary, but rather on an initiator (receptor) or effector of the studied pathways, and appearing to exert localized effects, particularly on thermogenesis. These results align with previous studies identifying the MT2 receptor as key in regulating insulin secretion and glucose metabolism (Sharma et al., 2015). Furthermore, the proximity of *MTNR1B* in the network to thermogenesis-related genes such as *UCP1*, *UCP3*, *PPARGC1A*, and *PPARG*, suggesting a dual role in regulating glucose metabolism and thermogenesis. These findings highlight *MTNR1B* as a promising therapeutic target for metabolic disorders. It is also striking that *TP53* was also identified in the list of diabesity-related genes (Table 5), as it is one of the most frequently mutated genes in cancer. This underscores the close relationship between obesity, its associated metabolic complications, circadian syndrome and an increased risk of cancer (Zwezdaryk et al., 2018). This is in line with previous results from our group and others in which melatonin inhibits tumor growth enhancing cancer prevention and treatment (Li et al., 2017; Agil et al., 2020; Zolfagharypoor et al., 2025). Moreover, sleep disturbances and circadian disruption have been related to cancer risk (Haus and Smolensky, 2013) maybe due to common pathways. Additionally, *SIRT1*, known for its role in mitochondrial biogenesis and adipogenesis, was identified as another key player, reinforcing its involvement in obesity progression (Majeed et al., 2021) and its reduced expression in the myocardium of diabetic patients (Du et al., 2024), further emphasizing its connection to diabesity.

In the present study, we analyze for the first time the impact of *MTNR1B* gene silencing in human myoblasts on melatonin-induced thermogenesis, which is previously demonstrated in the muscle from obese-diabetic rats, an *in vivo* model for the study of diabesity closely resembling human T2DM (Salagre et al., 2024b). Furthermore, the inhibition of MT2 protein expression was found to be lower than *MTNR1B* gene knockdown, suggesting that MT2 expression underlies post-transcriptional regulatory mechanism, that human myoblast have MT2 reservoirs, and/or MT2 protein has low protein turnover rate (Wu et al., 2004; Schwanhüusser et al., 2011; Vogel and Marcotte, 2012). This dose-dependent relationship in *MTNR1B* gene inhibition observed in this study may be useful in future research to optimize experimental design in gene silencing models, especially when aiming for effective inhibition without resorting to excessive siRNA concentrations, which could lead to unwanted side effects or cellular toxicity. These results provide evidence of the effectiveness of siRNA in reducing *MTNR1B* expression and its potential utility as a tool for studying the function of this gene in experimental models related to metabolic disorders, such as obesity and type 2 diabetes.

Here, the role of melatonin on the MT2 receptor was also investigated by evaluating its expression after the silencing of *MTNR1B*. The results provide evidence that melatonin significantly increases the expression of the MT2 receptor. It was observed that in the Scrambled siRNA negative controls, the expression of MT2 remained constant at concentrations of 40 nM and 120 nM, indicating a stable basal expression of the receptor even after transfection. However, when melatonin was added to the cells, the expression of MT2 increased significantly. This increase in MT2 expression may confirm that melatonin promotes the activation of this receptor, although direct assessment of downstream signaling pathways need to be further explored. Conversely, after the knockdown, melatonin addition showed no variations in MT2 expression compared to cells not treated with melatonin, suggesting that the functionality and proper expression of the MT2 receptor is essential for its positive regulation by melatonin. This indicates that the inhibition of *MTNR1B* reduces the ability of cells to respond to melatonin effectively and suggests that the functionality of MT2 is crucial for the observed melatonin effects in obese-diabetic rats.

Our previous study showed that melatonin increased muscle NST through SLN overexpression and SERCA uncoupling *via* Ca^2+^-dependent pathway activation (Salagre et al., 2024b). MT2 expression was found to be related to Ca^2+^ homeostasis in the skeletal muscle of obese-diabetic rats (Agil et al., 2015; Salagre et al., 2024a) and human myocytes (Sasaki et al., 2021), also being essential for SERCA2 expression in heart (Prado et al., 2020) and other tissues (Ren et al., 2024). Furthermore, MT2 was shown to be the key receptor for melatonin effects increasing lipolysis and thermogenesis (Tripathy and Bhattamisra, 2025). These results are coherent with those obtained in the present study in which MT2 functionality and expression was essential for increased SERCA1/2 and SLN expression by melatonin in human myoblast. Gene knockdown of *MTNR1B* reversed the observed melatonin effects recovering basal or even further decreasing SERCA1/2 and SLN expression. Moreover, decreased SERCA2 expression but no SERCA1 was observed in MT2 silenced human myoblast suggesting a closer relationship between MT2 and SERCA2 than SERCA1 in the thermogenic effects of melatonin. This association was also observed in our previous study showing SERCA2, but not SERCA1, overexpression after melatonin treatment in the muscle of obese-diabetic rats (Salagre et al., 2024b). SLN is an important protein involved in Ca^2+^-dependent muscle thermogenesis, and its function is closely related to the activation of the thermogenic pathway (Salagre et al., 2024b). In this study, a significant increase in SLN expression was observed in cells treated with melatonin, indicating that this hormone may promote SLN expression, which is consistent with previous research showing that melatonin has a positive effect on the expression of proteins involved in muscle thermogenesis (Aouichat et al., 2022; Salagre et al., 2024b). These results support the idea that melatonin can promote thermogenesis through the positive regulation of SLN and MT2, increasing SERCA activity uncoupling. Moreover, the increase in SERCA1/2 expression could also be explained as a compensatory mechanism to maintain calcium transport due to the reduced SERCA activity. Previous results from our team showed that melatonin increased both SERCA activity and expression in obese-diabetic rats supporting the close association between SERCA function and melatonin (Salagre et al., 2024b), although the lack of SERCA activity measurements in the present study could be a limitation and further research focusing the association of SERCA activity and MT2 knockdown is needed. Our results demonstrate that SLN expression was significantly reduced when the *MTNR1B* gene was silenced using 120 nM siRNA, highlighting the importance of effective MT2 silencing to achieve the expected effects on the expression of thermogenic proteins. The decrease in the expression SLN and SERCA2 in *MTNR1B* knockdown human myoblast reinforces the hypothesis that the activity of MT2 is crucial for metabolic function, particularly thermogenesis regulation. In the presence of melatonin, no recovery of SLN and SERCA1/2 expression was observed in the *MTNR1B* siRNA-transfected cells, indicating that the functionality of the MT2 receptor is crucial for melatonin-induced regulation of SERCA/SLN uncoupling and, therefore, for the activation of the Ca^2+^-dependent thermogenic pathway in which similar effects were found in CaMKII/AMPK/PGC1α activation. In human myoblasts with unchanged MT2 protein levels, P-CaMKII, P-AMPK, PGC1α and calcineurin expression were increased after melatonin treatment confirming the previously observed results in the muscle of obese-diabetic rats in which melatonin activated the Ca^2+^-dependent thermogenic pathway *via* SERCA/SLN uncoupling (Salagre et al., 2024b; 2025). Furthermore, melatonin slightly increased the phosphorylation levels of key thermogenic pathway mediators, including CaMKII, AMPK, and PGC1α, in *MTNR1B* silenced cells at 40 nM but not at 120 nM suggesting that only at 120 nM an effective gene knockdown was achieved as previously mentioned. Cells with effective *MTNR1B* gene knockdown, also presented decreased activation by phosphorylation of CaMKII and AMPK proteins and no effects after melatonin treatment showing the close relationship between MT2 functionality and the activation of Ca^2+^-dependent pathways. These results are coherent with previous works in pancreatic cells from rats that showed increased insulin secretion after melatonin treatment through MT2 receptor and a Ca^2+^-dependent pathway activation *via* CaMKII (Bazwinsky-Wutschke et al., 2012; 2014). Moreover, melatonin recovered the sleep phase delayed in mice *via* CaMKII regulation after MT2 activation (Wang et al., 2020). Similarly, studies *in vitro* in mammalian reproductive endocrine cells showed that melatonin regulates progesterone/testosterone production through an AMPK-mediated pathway *via* MT2 activation promoting mitochondrial function (Zhao et al., 2024) and autophagy (Duan et al., 2024). AMPK was also shown to be a key regulator of melatonin thermogenic effects in obese-diabetic rats enhancing skeletal muscle mitochondria biogenesis (Salagre et al., 2024b), function (Salagre et al., 2025), and autophagy (Salagre et al., 2023), reducing muscle organellar stress (Salagre et al., 2024a).

In conclusion, the present study provides compelling clear evidence that the MT2 receptor encoded by *MTNR1B*, plays, at least partially, a pivotal role in melatonin-induced skeletal muscle NST by promoting SERCA uncoupling by SLN upregulation and activating Ca^2+^-dependent thermogenic pathways *via* CaMKII/AMPK/PGC1α signaling. These findings position melatonin as an effective and safe therapy against diabesity and circadian syndrome by enhancing thermogenic activation, however, further in-depth functional assay studies are needed to corroborate the direct thermogenic effect of melatonin on human myoblasts *via* the MT2 receptor. Future research should focus on further elucidating the role of MT2 and MT1 polymorphisms in the thermogenic effects of melatonin in humans, and investigating other melatonin receptors, such as MT1 and its gene *MTNR1A*, to gain a comprehensive understanding of melatonin’s role in metabolic regulation and the involvement of its receptor expression, being this a limitation of the present study. This study lays the groundwork for developing novel melatonin-based therapies to combat obesity and its associated type 2 diabetes.

## Data Availability

The original contributions presented in the study are included in the article/Supplementary Material, further inquiries can be directed to the corresponding author.

## References

[B1] Agil A. Benhaj K. Navarro-Alarcon M. Abdo W. Zourgui L. Entrena J. M. (2020). Melatonin inhibits growth of B16 melanoma in C57BL/6 mice. Melat. Res. 3, 436–450. 10.32794/MR11250071

[B2] Agil A. Elmahallawy E. K. Rodríguez-Ferrer J. M. Adem A. Bastaki S. M. Al-Abbadi I. (2015). Melatonin increases intracellular calcium in the liver, muscle, white adipose tissues and pancreas of diabetic obese rats. Food Funct. 6, 2671–2678. 10.1039/C5FO00590F 26134826

[B3] Agil A. Navarro-Alarcon M. Ali F. A. Z. Albrakati A. Salagre D. Campoy C. (2021). Melatonin enhances the mitochondrial functionality of brown adipose tissue in obese-diabetic rats. Antioxidants 10, 1482. 10.3390/ANTIOX10091482 34573114 PMC8466890

[B4] Agil A. Navarro-Alarcõn M. Ruiz R. Abuhamadah S. El-Mir M. Y. Vázquez G. F. (2011). Beneficial effects of melatonin on obesity and lipid profile in young Zucker diabetic fatty rats. J. Pineal Res. 50, 207–212. 10.1111/J.1600-079X.2010.00830.X 21087312

[B5] Agil A. Rosado I. Ruiz R. Figueroa A. Zen N. Fernández-Vázquez G. (2012). Melatonin improves glucose homeostasis in young Zucker diabetic fatty rats. J. Pineal Res. 52, 203–210. 10.1111/J.1600-079X.2011.00928.X 21883445

[B6] Aouichat S. Raya E. Molina-Carballo A. Munoz-Hoyos A. Aloweidi A. S. Elmahallawy E. K. (2022). Dose-dependent effect of melatonin on BAT thermogenesis in Zücker diabetic fatty rat: future clinical implications for obesity. Antioxidants 11, 1646. 10.3390/ANTIOX11091646 36139720 PMC9495691

[B7] Azzeh F. S. Kamfar W. W. Ghaith M. M. Alsafi R. T. Shamlan G. Ghabashi M. A. (2024). Unlocking the health benefits of melatonin supplementation: a promising preventative and therapeutic strategy. Medicine 103, e39657. 10.1097/MD.0000000000039657 39312371 PMC11419438

[B8] Bazwinsky-Wutschke I. Mühlbauer E. Albrecht E. Peschke E. (2014). Calcium-signaling components in rat insulinoma β-cells (INS-1) and pancreatic islets are differentially influenced by melatonin. J. Pineal Res. 56, 439–449. 10.1111/JPI.12135 24650091

[B9] Bazwinsky-Wutschke I. Wolgast S. Mühlbauer E. Albrecht E. Peschke E. (2012). Phosphorylation of cyclic AMP-response element-binding protein (CREB) is influenced by melatonin treatment in pancreatic rat insulinoma β-cells (INS-1). J. Pineal Res. 53, 344–357. 10.1111/J.1600-079X.2012.01004.X 22616931

[B10] Capcarova M. Kalafova A. (2019). “Zucker diabetic fatty rats for research in diabetes,” in Animal models in medicine and biology. Editors E. Tvrdá and S. C. Yenisetti (London: IntechOpen). 10.5772/INTECHOPEN.88161

[B11] Challet E. Pévet P. (2024). Melatonin in energy control: circadian time-giver and homeostatic monitor. J. Pineal Res. 76, e12961. 10.1111/JPI.12961 38751172

[B12] Chen B. You W. Shan T. (2019). Myomaker, and Myomixer-Myomerger-Minion modulate the efficiency of skeletal muscle development with melatonin supplementation through Wnt/β-catenin pathway. Exp. Cell Res. 385, 111705. 10.1016/J.YEXCR.2019.111705 31682812

[B13] Cheng M. Liu X. Yang M. Han L. Xu A. Huang Q. (2017). Computational analyses of type 2 diabetes-associated loci identified by genome-wide association studies. J. Diabetes 9, 362–377. 10.1111/1753-0407.12421 27121852

[B14] Cipolla-Neto J. Amaral F. G. Afeche S. C. Tan D. X. Reiter R. J. (2014). Melatonin, energy metabolism, and obesity: a review. J. Pineal Res. 56, 371–381. 10.1111/JPI.12137 24654916

[B15] Delpino F. M. Figueiredo L. M. (2021). Melatonin supplementation and anthropometric indicators of obesity: a systematic review and meta-analysis. Nutrition 91–92, 111399. 10.1016/J.NUT.2021.111399 34626955

[B16] Doghramji K. (2007). Melatonin and its receptors: a new class of sleep-promoting agents. J. Clin. Sleep Med. 3, S17–S23. 10.5664/jcsm.26932 17824497 PMC1978320

[B17] Du Z. Zhou Y. Li Q. Xie Y. Zhu T. Qiao J. (2024). SIRT1 ameliorates lamin A/C deficiency-induced cardiac dysfunction by promoting mitochondrial bioenergetics. JACC Basic Transl. Sci. 9, 1211–1230. 10.1016/J.JACBTS.2024.05.011 39534638 PMC11551877

[B18] Duan H. Yang S. Xiao L. Yang S. Yan Z. Wang F. (2024). Melatonin promotes progesterone secretion in sheep luteal cells by regulating autophagy *via* the AMPK/mTOR pathway. Theriogenology 214, 342–351. 10.1016/J.THERIOGENOLOGY.2023.11.010 37976799

[B19] Emet M. Ozcan H. Ozel L. Yayla M. Halici Z. Hacimuftuoglu A. (2016). A review of melatonin, its receptors and drugs. Eurasian J. Med. 48, 135–141. 10.5152/EURASIANJMED.2015.0267 27551178 PMC4970552

[B20] Fernández Vázquez G. Reiter R. J. Agil A. (2018). Melatonin increases brown adipose tissue mass and function in Zücker diabetic fatty rats: implications for obesity control. J. Pineal Res. 64, e12472. 10.1111/JPI.12472 29405372

[B21] Gao X. Sun H. Wei Y. Niu J. Hao S. Sun H. (2024). Protective effect of melatonin against metabolic disorders and neuropsychiatric injuries in type 2 diabetes mellitus mice. Phytomedicine 131, 155805. 10.1016/J.PHYMED.2024.155805 38851097

[B22] Haus E. L. Smolensky M. H. (2013). Shift work and cancer risk: potential mechanistic roles of circadian disruption, light at night, and sleep deprivation. Sleep. Med. Rev. 17, 273–284. 10.1016/J.SMRV.2012.08.003 23137527

[B23] Hong S. H. Kim J. (2024). Melatonin and metabolic disorders: unraveling the interplay with glucose and lipid metabolism, adipose tissue, and inflammation. Sleep. Med. Rev. 15, 70–80. 10.17241/SMR.2024.02159

[B24] Izzo G. Francesco A. Ferrara D. Campitiello M. R. Serino I. Minucci S. (2010). Expression of melatonin (MT1, MT2) and melatonin-related receptors in the adult rat testes and during development. Zygote 18, 257–264. 10.1017/S0967199409990293 20109269

[B25] Jiménez-Aranda A. Fernández-Vázquez G. Campos D. Tassi M. Velasco-Perez L. Tan D. X. (2013). Melatonin induces browning of inguinal white adipose tissue in Zucker diabetic fatty rats. J. Pineal Res. 55, 416–423. 10.1111/JPI.12089 24007241

[B26] Jura M. Kozak L. P. (2016). Obesity and related consequences to ageing. Age (Dordr) 38, 23. 10.1007/S11357-016-9884-3 26846415 PMC5005878

[B27] Kim C. H. Kim K. H. Yoo Y. M. (2012). Melatonin-induced autophagy is associated with degradation of MyoD protein in C2C12 myoblast cells. J. Pineal Res. 53, 289–297. 10.1111/j.1600-079X.2012.00998.x 22582971

[B28] Kolberg L. Raudvere U. Kuzmin I. Adler P. Vilo J. Peterson H. (2023). G:Profiler-interoperable web service for functional enrichment analysis and gene identifier mapping (2023 update). Nucleic Acids Res. 51, W207–W212. 10.1093/nar/gkad347 37144459 PMC10320099

[B29] Koziróg M. Poliwczak A. R. Duchnowicz P. Koter-Michalak M. Sikora J. Broncel M. (2011). Melatonin treatment improves blood pressure, lipid profile, and parameters of oxidative stress in patients with metabolic syndrome. J. Pineal Res. 50, 261–266. 10.1111/J.1600-079X.2010.00835.X 21138476

[B30] Li Q. Zhang S. Wang H. Wang Z. Zhang X. Wang Y. (2023). Association of rotating night shift work, CLOCK, MTNR1A, MTNR1B genes polymorphisms and their interactions with type 2 diabetes among steelworkers: a case–control study. BMC Genomics 24, 232. 10.1186/S12864-023-09328-Y 37138267 PMC10157991

[B31] Li Y. Li S. Zhou Y. Meng X. Zhang J. J. Xu D. P. (2017). Melatonin for the prevention and treatment of cancer. Oncotarget 8, 39896–39921. 10.18632/ONCOTARGET.16379 28415828 PMC5503661

[B32] Liu J. Clough S. J. Hutchinson A. J. Adamah-Biassi E. B. Popovska-Gorevski M. Dubocovich M. L. (2016). MT1 and MT2 melatonin receptors: a therapeutic perspective. Annu. Rev. Pharmacol. Toxicol. 56, 361–383. 10.1146/ANNUREV-PHARMTOX-010814-124742 26514204 PMC5091650

[B33] Luo Z. Tang Y. Y. Zhou L. (2024). Melatonin as an adjunctive therapy in cardiovascular disease management. Sci. Prog. 107, 368504241299993. 10.1177/00368504241299993 39574322 PMC11585022

[B34] Lyssenko V. Nagorny C. L. F. Erdos M. R. Wierup N. Jonsson A. Spégel P. (2009). Common variant in MTNR1B associated with increased risk of type 2 diabetes and impaired early insulin secretion. Nat. Genet. 41, 82–88. 10.1038/NG.288 19060908 PMC3725650

[B35] Ma H. Kang J. Fan W. He H. Huang F. (2021). ROR: nuclear receptor for melatonin or not? Molecules 26, 2693. 10.3390/MOLECULES26092693 34064466 PMC8124216

[B36] Majeed A. Mukhtar S. (2023). Protein-protein interaction network exploration using cytoscape. Methods Mol. Biol. 2690, 419–427. 10.1007/978-1-0716-3327-4_32 37450163

[B37] Majeed Y. Halabi N. Madani A. Y. Engelke R. Bhagwat A. M. Abdesselem H. (2021). SIRT1 promotes lipid metabolism and mitochondrial biogenesis in adipocytes and coordinates adipogenesis by targeting key enzymatic pathways. Sci. Rep. 11, 8177. 10.1038/S41598-021-87759-X 33854178 PMC8046990

[B38] Morvaridzadeh M. Sadeghi E. Agah S. Nachvak S. M. Fazelian S. Moradi F. (2020). Effect of melatonin supplementation on oxidative stress parameters: a systematic review and meta-analysis. Pharmacol. Res. 161, 105210. 10.1016/J.PHRS.2020.105210 33007423

[B39] Navarro-Alarcón M. Ruiz-Ojeda F. J. Blanca-Herrera R. M. A-Serrano M. M. Acuña-Castroviejo D. Fernández-Vázquez G. (2014). Melatonin and metabolic regulation: a review. Food Funct. 5, 2806–2832. 10.1039/C4FO00317A 25207999

[B40] Nikolaev G. Robeva R. Konakchieva R. (2021). Membrane melatonin receptors activated cell signaling in physiology and disease. Int. J. Mol. Sci. 23, 471. 10.3390/IJMS23010471 35008896 PMC8745360

[B41] Owino S. Buonfiglio D. D. C. Tchio C. Tosini G. (2019). Melatonin signaling a key regulator of glucose homeostasis and energy metabolism. Front. Endocrinol. (Lausanne) 10, 488. 10.3389/FENDO.2019.00488 31379753 PMC6651071

[B42] Owino S. Contreras-Alcantara S. Baba K. Tosini G. (2016). Melatonin signaling controls the daily rhythm in blood glucose levels independent of peripheral clocks. PLoS One 11, e0148214. 10.1371/JOURNAL.PONE.0148214 26824606 PMC4732609

[B43] Panmanee J. Charoensutthivarakul S. Cheng C. W. Promthep K. Mukda S. Prasertporn T. (2025). A complex interplay between melatonin and RORβ: rorβ is unlikely a putative receptor for melatonin as revealed by biophysical assays. Mol. Neurobiol. 62, 2333–2347. 10.1007/S12035-024-04395-Y 39105871 PMC11772548

[B44] Prado N. J. Muñoz E. M. Altamirano L. E. F. Aguiar F. Zumino A. Z. P. Sánchez F. J. (2020). Reperfusion arrhythmias increase after superior cervical ganglionectomy due to conduction disorders and changes in repolarization. Int. J. Mol. Sci. 21, 1–16. 10.3390/IJMS21051804 PMC7084297 32155697

[B45] Promsan S. Lungkaphin A. (2020). The roles of melatonin on kidney injury in obese and diabetic conditions. BioFactors 46, 531–549. 10.1002/BIOF.1637 32449276

[B46] Raudvere U. Kolberg L. Kuzmin I. Arak T. Adler P. Peterson H. (2019). g:Profiler: a web server for functional enrichment analysis and conversions of gene lists (2019 update). Nucleic Acids Res. 47, W191–W198. 10.1093/NAR/GKZ369 31066453 PMC6602461

[B47] Reimand J. Isserlin R. Voisin V. Kucera M. Tannus-Lopes C. Rostamianfar A. (2019). Pathway enrichment analysis and visualization of omics data using g:Profiler, GSEA, Cytoscape and EnrichmentMap. Nat. Protoc. 14, 482–517. 10.1038/S41596-018-0103-9 30664679 PMC6607905

[B48] Ren C. Hu C. Hu M. Wu Y. Yang Y. Lu F. (2024). Melatonin protects RPE cells from necroptosis and NLRP3 activation *via* promoting SERCA2-related intracellular Ca^2+^ homeostasis. Phytomedicine 135, 156088. 10.1016/J.PHYMED.2024.156088 39341129

[B49] Saklayen M. G. (2018). The global epidemic of the metabolic syndrome. Curr. Hypertens. Rep. 20, 12. 10.1007/S11906-018-0812-Z 29480368 PMC5866840

[B50] Salagre D. Bajit H. Fernández-Vázquez G. Dwairy M. Garzón I. Haro-López R. (2025). Melatonin induces fiber switching by improvement of mitochondrial oxidative capacity and function *via* NRF2/RCAN/MEF2 in the vastus lateralis muscle from both sex Zücker diabetic fatty rats. Free Radic. Biol. Med. 227, 322–335. 10.1016/J.FREERADBIOMED.2024.12.019 39645208

[B51] Salagre D. Chayah M. Molina-Carballo A. Oliveras-López M. J. Munoz-Hoyos A. Navarro-Alarcón M. (2022). Melatonin induces fat browning by transdifferentiation of white adipocytes and *de novo* differentiation of mesenchymal stem cells. Food Funct. 13, 3760–3775. 10.1039/D1FO04360A 35274657

[B52] Salagre D. Navarro-Alarcón M. González L. G. Elrayess M. A. Villalón-Mir M. Haro-López R. (2024a). Melatonin ameliorates organellar calcium homeostasis, improving endoplasmic reticulum stress-mediated apoptosis in the vastus lateralis muscle of both sexes of obese diabetic rats. Antioxidants 14, 16. 10.3390/ANTIOX14010016 39857351 PMC11762543

[B53] Salagre D. Navarro-Alarcón M. Villalón-Mir M. Alcázar-Navarrete B. Gómez-Moreno G. Tamimi F. (2024b). Chronic melatonin treatment improves obesity by inducing uncoupling of skeletal muscle SERCA-SLN mediated by CaMKII/AMPK/PGC1α pathway and mitochondrial biogenesis in female and male Zücker diabetic fatty rats. Biomed. Pharmacother. 172, 116314. 10.1016/J.BIOPHA.2024.116314 38387135

[B54] Salagre D. Raya Álvarez E. Cendan C. M. Aouichat S. Agil A. (2023). Melatonin improves skeletal muscle structure and oxidative phenotype by regulating mitochondrial dynamics and autophagy in Zücker diabetic fatty rat. Antioxidants (Basel) 12, 1499. 10.3390/ANTIOX12081499 37627494 PMC10451278

[B55] Sasaki H. Zhang Y. Emala C. W. Mizuta K. (2021). Melatonin MT2 receptor is expressed and potentiates contraction in human airway smooth muscle. Am. J. Physiol. Lung Cell Mol. Physiol. 321, L991–L1005. 10.1152/AJPLUNG.00273.2021 34612067 PMC8715028

[B56] Schwanhüusser B. Busse D. Li N. Dittmar G. Schuchhardt J. Wolf J. (2011). Global quantification of mammalian gene expression control. Nature 473, 337–342. 10.1038/NATURE10098 21593866

[B57] Sharma S. Singh H. Ahmad N. Mishra P. Tiwari A. (2015). The role of melatonin in diabetes: therapeutic implications. Arch. Endocrinol. Metab. 59, 391–399. 10.1590/2359-3997000000098 26331226

[B58] Shiota M. Printz R. L. (2012). Diabetes in Zucker diabetic fatty rat. Methods Mol. Biol. 933, 103–123. 10.1007/978-1-62703-068-7_8 22893404

[B59] Slominski R. M. Reiter R. J. Schlabritz-Loutsevitch N. Ostrom R. S. Slominski A. T. (2012). Melatonin membrane receptors in peripheral tissues: distribution and functions. Mol. Cell Endocrinol. 351, 152–166. 10.1016/J.MCE.2012.01.004 22245784 PMC3288509

[B60] Tan D. X. Manchester L. C. Qin L. Reiter R. J. (2016). Melatonin: a mitochondrial targeting molecule involving mitochondrial protection and dynamics. Int. J. Mol. Sci. 17, 2124. 10.3390/IJMS17122124 27999288 PMC5187924

[B61] Tripathy S. Bhattamisra S. K. (2025). Cellular signalling of melatonin and its role in metabolic disorders. Mol. Biol. Rep. 52, 193. 10.1007/S11033-025-10306-8 39903334

[B62] Vogel C. Marcotte E. M. (2012). Insights into the regulation of protein abundance from proteomic and transcriptomic analyses. Nat. Rev. Genet. 13, 227–232. 10.1038/NRG3185 22411467 PMC3654667

[B63] Waly N. E. Hallworth R. (2015). Circadian pattern of melatonin MT1 and MT2 receptor localization in the rat suprachiasmatic nucleus. J. Circadian Rhythms 13, 1–7. 10.5334/JCR.AB 27103927 PMC4831275

[B64] Wang F. (2021). Semi-quantitative RT-PCR: an effective method to explore the regulation of gene transcription level affected by environmental pollutants. Methods Mol. Biol. 2326, 95–103. 10.1007/978-1-0716-1514-0_7 34097263

[B65] Wang Q. Zhu D. Ping S. Li C. Pang K. Zhu S. (2020). Melatonin recovers sleep phase delayed by MK-801 through the melatonin MT2 receptor- Ca^2+^ -CaMKII-CREB pathway in the ventrolateral preoptic nucleus. J. Pineal Res. 69, e12674. 10.1111/JPI.12674 32535982

[B66] Wang T. Wang X. T. Lai R. Ling H. W. Zhang F. Lu Q. (2019). MTNR1B gene polymorphisms are associated with the therapeutic responses to repaglinide in Chinese patients with type 2 diabetes mellitus. Front. Pharmacol. 10, 1318. 10.3389/FPHAR.2019.01318 31787898 PMC6855210

[B67] Wilkin T. J. Voss L. D. (2004). Metabolic syndrome: maladaptation to a modern world. J. R. Soc. Med. 97, 511–520. 10.1177/014107680409701102 15520144 PMC1079643

[B68] World Health Organization (2022). Obesity and overweight. Available online at: https://www.who.int/news-room/fact-sheets/detail/obesity-and-overweight (Accessed March 10, 2025).

[B69] Wu W. Hodges E. Redelius J. Höög C. (2004). A novel approach for evaluating the efficiency of siRNAs on protein levels in cultured cells. Nucleic Acids Res. 32, e17. 10.1093/NAR/GNH010 14739231 PMC373369

[B70] Xu Z. You W. Liu J. Wang Y. Shan T. (2020). Elucidating the regulatory role of melatonin in Brown, white, and beige adipocytes. Adv. Nutr. 11, 447–460. 10.1093/ADVANCES/NMZ070 31355852 PMC7442421

[B71] Zhang K. Ma Y. Luo Y. Song Y. Xiong G. Ma Y. (2023). Metabolic diseases and healthy aging: identifying environmental and behavioral risk factors and promoting public health. Front. Public Health 11, 1253506. 10.3389/FPUBH.2023.1253506 37900047 PMC10603303

[B72] Zhang Y. Yang Y. Sun Y. Wei Z. Wang D. Chen S. (2025). Assessing the toxicological impact of PET-MPs exposure on IVDD: insights from network toxicology and molecular docking. J. Environ. Manage 373, 123830. 10.1016/J.JENVMAN.2024.123830 39736229

[B73] Zhao Y. Qin G. Jiang B. Huang J. He S. Peng H. (2024). Melatonin regulates mitochondrial function to alleviate ferroptosis through the MT2/Akt signaling pathway in swine testicular cells. Sci. Rep. 14, 15215. 10.1038/S41598-024-65666-1 38956409 PMC11219911

[B74] Zimmet P. Alberti K. G. M. M. Stern N. Bilu C. El-Osta A. Einat H. (2019). The Circadian syndrome: is the metabolic syndrome and much more. J. Intern Med. 286, 181–191. 10.1111/JOIM.12924 31081577 PMC6851668

[B75] Zolfagharypoor A. Ajdari A. Seirafianpour F. Pakbaz Y. Hosseinzadeh A. Mehrzadi S. (2025). Signaling pathways in skin cancers and the protective functions of melatonin. Biochimie 231, 1–14. 10.1016/J.BIOCHI.2024.11.013 39577617

[B76] Zwezdaryk K. Sullivan D. Saifudeen Z. (2018). The p53/adipose-tissue/cancer nexus. Front. Endocrinol. (Lausanne) 9, 457. 10.3389/FENDO.2018.00457 30158901 PMC6104444

